# Cytotoxic NK Cells Impede Response to Checkpoint Immunotherapy in Melanoma with an Immune-Excluded Phenotype

**DOI:** 10.1158/2159-8290.CD-24-1208

**Published:** 2025-06-18

**Authors:** Joanna Pozniak, Niccolò Roda, Ewout Landeloos, Asier Antoranz, Yannick Van Herck, Amber De Visscher, Philip Georg Demaerel, Lukas Vanwynsberghe, Jeroen Declercq, Christos Gkemisis, Greet Bervoets, No-Joon Song, Ayse Bassez, Robin Browaeys, Lotte Pollaris, Francesca M. Bosisio, Veerle Boecxstaens, Yvan Saeys, Diether Lambrechts, Zihai Li, Patrick Matthys, Oliver Bechter, Jean-Christophe Marine

**Affiliations:** 1 Laboratory for Molecular Cancer Biology, Center for Cancer Biology, VIB, Leuven, Belgium.; 2 Laboratory for Molecular Cancer Biology, Department of Oncology, KU Leuven, Leuven, Belgium.; 3 Department of General Medical Oncology, UZ Leuven, Leuven, Belgium.; 4 Laboratory of Translational Cell and Tissue Research, Department of Pathology, KU Leuven, Leuven, Belgium.; 5 Laboratory of Immunobiology, Department of Microbiology, Immunology and Transplantation, Rega Institute, KU Leuven, Leuven, Belgium.; 6 Department of Dermatology, UZ Leuven, Leuven, Belgium.; 7 Pelotonia Institute for Immuno-Oncology, The Ohio State University Comprehensive Cancer Center- James Cancer Center and Solove Research Institute, Columbus, Ohio.; 8 Laboratory of Translational Genetics, Center for Cancer Biology, VIB, Leuven, Belgium.; 9 Center for Human Genetics, KU Leuven, Leuven, Belgium.; 10 Data Mining and Modelling for Biomedicine, VIB Center for Inflammation Research, Ghent, Belgium.; 11 Department of Applied Mathematics, Computer Science and Statistics, Ghent University, Ghent, Belgium.; 12 Department of Pathology, UZLeuven, Leuven, Belgium.; 13 Department of Surgical Oncology, UZLeuven, Leuven, Belgium.; 14 Division of Medical Oncology, Department of Internal Medicine, The Ohio State University, Columbus, Ohio.

## Abstract

Immune checkpoint blockade (ICB) has revolutionized cancer treatment. Unfortunately, the inability of lymphocytes to infiltrate the tumor nest, a phenomenon known as immune exclusion, drastically limits ICB responsiveness. Analyzing the immune landscape of matched pre- and early on-treatment biopsies of patients with melanoma undergoing ICB therapy, we observed a significant increase in cytotoxic NK cells in early on-treatment biopsies from nonresponders. Spatial multiomic analyses revealed that, although NK cells colocalized with CD8^+^ T cells within the tumor bed in responding lesions, they were excluded from the tumor parenchyma in nonresponding lesions. Strikingly, pharmacologic depletion of NK cells in a unique melanoma mouse model exhibiting an immune-excluded phenotype unleashed immune infiltration of the tumor core and tumor clearance upon ICB exposure. Mechanistically, we show that NK cells are actively recruited to immune-excluded areas upon ICB exposure via the chemokine receptor CX3CR1 to suppress tumor infiltration and antitumor function of CD8^+^ T cells.

**Significance:**

Immune exclusion is responsible for intrinsic resistance to ICB in about half of nonresponder patients. Our unexpected observation that targeting NK cell biology unleashes the recruitment and antitumor activity of CD8^+^ T cells in tumors with an immune-excluded phenotype offers a potential therapeutic avenue for this large patient population.

*See related commentary by Galvez-Cancino et al., p. 1777*

*See related article by Song et al., p. 1835*

## Teaser

Cytotoxic NK cells accumulate at tumor margins in a CX3CR1-dependent manner following exposure to immune checkpoint blockade (ICB), and NK cell depletion enhances CD8^+^ T-cell infiltration into melanoma and overcomes ICB resistance.

## Introduction

Despite the clinical success of immune checkpoint blockade (ICB), about half of patients with metastatic melanoma do not respond. Biomarkers to predict response are needed, and understanding resistance mechanisms is key to improving treatment success (1). Immune exclusion, the failure of lymphocytes to infiltrate tumors, often predicts resistance to ICB, although the mechanisms underlying this phenomenon remain unclear.

NK cells are known as crucial effectors of antitumor immune responses (2–5), albeit with recent studies challenging this paradigm. Tumor-associated CD69^+^ perforin^–^ NK cells were found to attract myeloid-derived suppressor cells in ICB-refractory tumors (6). In addition, NKp30^+^ NK cells were found to promote cytolysis of B7H6^+^ activated T cells in nonresponding ICB patients (7), whereas tumor-infiltrating dysfunctional CD56^dim^CD16^hi^ NK cells (expressing high levels of NR4A1 and inhibitory receptors and low levels of cytotoxic granules) were associated with resistance to immunotherapy (8). Although these findings suggest that NK cells may adopt a range of phenotypes and play intricate roles in influencing responses to ICB, it remains to be elucidated whether they directly contribute to therapy resistance and, if so, under which specific context this occurs.

Most melanoma studies on immune cells and ICB response have used samples from patients resistant to other treatments although ICB is now the first line. Additionally, these studies often analyzed biopsies taken before ICB treatment or at inconsistent, late stages (9). This is unfortunate because emerging evidence indicates that samples taken early during treatment are likely to be more predictive than pretreatment biopsies (10). Moreover, although immune cell topography in solid tumors is a key predictor for immunotherapy response (11, 12), most studies to date were performed on dissociated tumor samples and therefore lack spatial context. We reasoned that a single-cell, spatial analysis of melanoma biopsies from drug-naïve patients before and during ICB therapy could reveal insights into resistance mechanisms and guide the development of combination therapies or biomarkers.

## Results

### Dissecting the Immune Compartment of the Melanoma Ecosystem

**Figure 1.**
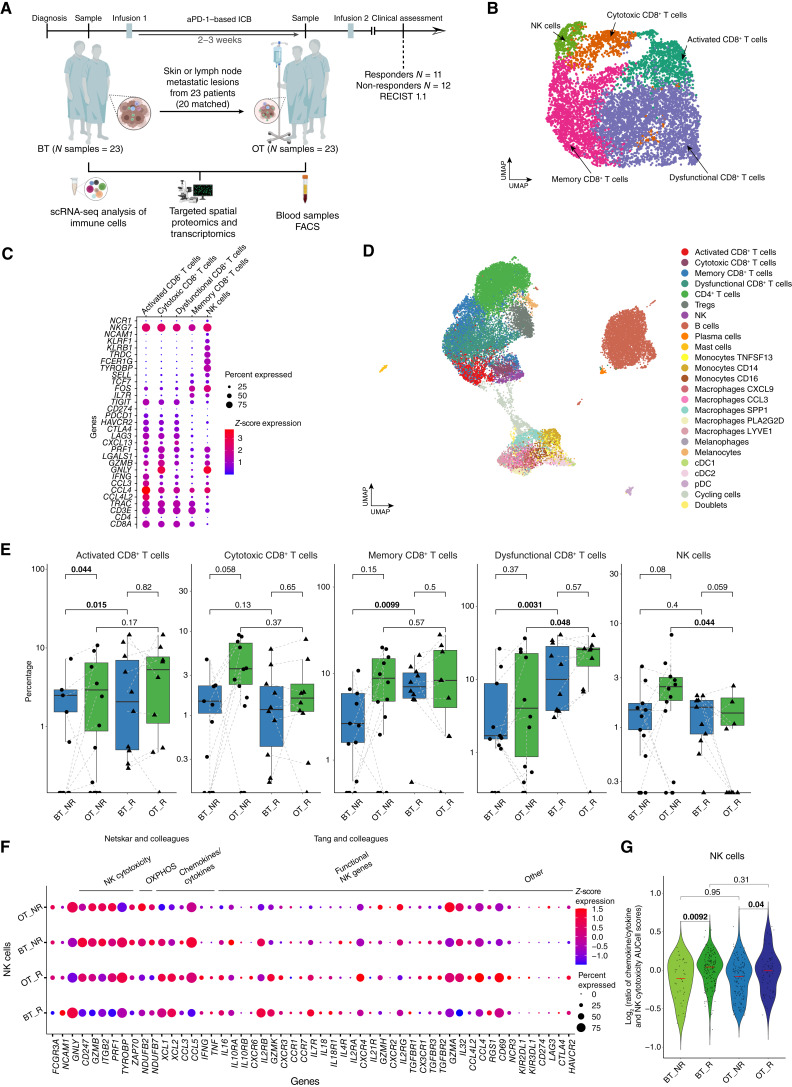
Dissecting the immune compartment of the melanoma ecosystem under immunotherapy pressure. **A,** Schematic describing the design of the SPECIAL clinical study, the timing of sample collection, number of patients enrolled, and samples collected, and single-cell (spatial) methodologies deployed for their in-depth characterization. **B,** UMAP of the CD8^+^ T cells and NK cells identified by unsupervised Louvain clustering of the subset of CD8^+^ T/NK cells including samples from both time points. **C,** Dotplot of the top representative discriminatory marker genes of the cells presented in **B** including samples from both time points. Size of the dot represents the number of cells expressing the gene and color reflects the expression level. **D,** UMAP of all immune cell types and states identified by summarizing unsupervised Louvain clustering and subclustering of myeloid and CD8^+^T/NK cells including samples from both time points. **E,** Percentage of CD8^+^ T-cell subsets and NK cells of all immune cells compared among Before treatment (BT) and On treatment (OT) samples and both response groups (two-sided Wilcoxon test, bold text points to significant differences = *P* < 0.05). **F,** Dotplot showing expression of NK-related genes, oxidative phosphorylation (OXPHOS), and chemokines/cytokine genes (highly discriminating group 1, 2, and 3 of NK cells) acquired from Netskar and colleagues (29) and NK functional genes acquired from Tang and colleagues (8) and other genes within NK cell population across response and time point groups. Size of the dot represents the number of cells expressing the gene and color reflects the expression level. **G,** Violin plot showing log_2_-transformed gene score (AUCell) ratios (chemokine/cytokine signature vs. NK cytotoxicity signature), calculated based on genes shown in **E**. cDC1, conventional type 1 DCs; cDC2, conventional type 2 DCs; R, responder; NR, Non-responder; UMAP, Uniform Manifold Approximation and Projection. (**A,** Created with BioRender.com.)

Biopsies (skin/cutaneous, *n* = 12; subcutaneous, *n* = 8; and lymph node, *n* = 26) were collected before treatment (BT) and early (± 2–3 weeks) on treatment (OT) from a cohort of treatment-naïve (stage III–IV) patients with metastatic melanoma (SPECIAL trial, #S62275; ref. 13). These patients received either anti–PD-1 (αPD-1) monotherapy (nivolumab or pembrolizumab, *n* = 17) or αPD-1 in combination with anti–CTLA4 (αCTLA4; ipilimumab + nivolumab, *n* = 6). A total of 20 matched biopsies were obtained from 23 patients. Patient clinical characteristics were obtained at baseline, and response to ICB was assessed using RECIST v1.1 criteria 3 months OT (Supplementary Table S1A). Fresh tissue was processed for single-cell RNA sequencing (scRNA-seq) and fixed material for pathologic assessment and spatial multiomic analyses (Fig. 1A). Peripheral blood samples were collected from all patients and used for immunophenotyping by flow cytometry (FACS).

After quality control and data normalization and integration, we identified 25,401 single immune cells based on high immune gene set activity (14) and low mean copy-number variant score (15). One sample was discarded because it contained less than 10 immune cells. Unsupervised Louvain clustering identified 17 immune cell clusters, which were annotated based on previously published cell type–specific gene signatures (16), resulting in 10 main immune cell types. These included CD8^+^ and CD4^+^ T cells, regulatory T cells, CD8^+^ T cells/NK cells, B cells, plasma cells, myeloid cells, mast cells, and plasmacytoid dendritic cells (pDC; Supplementary Fig. S1A and S1B; Supplementary Table S2). We identified a minor population of cells expressing high levels of pigment production genes (*MLANA* and *PMEL*) and genes involved in extracellular vesicle formation (e.g., *CD63* and *TIMP3*; Supplementary Table S2). These cells were predominantly detected in skin biopsies and annotated as melanocytes. Note that melanocytes express several innate immune genes (17), which explains why they had not been filtered out.

Cells from the CD8^+^ T cell/NK cell and myeloid cell clusters were further subclustered. In the CD8^+^ T-cell/NK cell cluster, 55% of cells were positive for *CD8A*, whereas 92% of cells expressed *GNLY*, suggesting that both cytotoxic T cells and cytotoxic NK cells populate this cluster (Supplementary Table S2). We therefore re-clustered all CD8^+^ T-cell clusters, thus retrieving a separate NK cell cluster characterized by high expression of KLRF1, *TYROBP*, *TRDC*, *NCAM1* (CD56), *NCR1*, and *NKG7* and low expression of *CD8A*, *CD4*, and *CD3E* (Fig. 1B and C; Supplementary Table S3). The cells from this cluster displayed a cytotoxic phenotype, with high expression of *GNLY*, *GZMB*, and *PRF1*, ruling out overlap with other innate lymphoid cell populations (18). CD8^+^ T cells were subdivided into memory (*IL7R*, *FOS*, *TCF7*, and *SELL*), activated (*IFNG*, *CCL3*, and *CCL4*), cytotoxic (*GNLY*, *GZMB*, and *PRF1*), and dysfunctional (expressing higher levels of *CXCL13*, *LAG3*, and *CTLA4* and lower levels of cytotoxic genes and *IFNG*; ref. 19).

As members of the mononuclear phagocyte system display transcriptional similarities, we re-clustered the myeloid and pDC cluster together, resulting in five subclusters: macrophages_*C1Q*, macrophages_*SPP1*, monocytes, dendritic cells (DC), and pDCs (Supplementary Fig. S1C; Supplementary Table S4). However, the macrophage_*C1Q* cluster contained cells expressing genes associated with an anti-inflammatory (*C1Q*, *APOE*, and *MRC1*) and a pro-inflammatory role (*CLXL9* and HLA genes). We re-clustered these cells into five subclusters: macrophages_*LYVE1*, macrophages_*CXCL9*, macrophages_*PLAG2D*, macrophages_*CCL3*, and melanophages (Supplementary Fig. S1D; Supplementary Table S5). The macrophages_*LYVE1* cluster largely overlapped with a known perivascular, M2-like phenotype, expressing both vascular growth factors involved in angiogenesis (*CD209*, *LILRB5*, and *PDGFC*) and genes associated with M2 polarization (*CD163*, *CSF1R*, and *MRC1*; Supplementary Table S5). The macrophages_*CXCL9* and macrophages_*CCL3* gene signatures overlapped with the signature of M1-like macrophages. Note that rather than being correlated with conventional M1 and M2 markers, the polarity of tumor-associated macrophages was associated with divergent expression in *CXCL9* and *CXCL10* versus *SPP1* (20). The macrophage_*CCL3* cluster was characterized by both proinflammatory cytokines (*CCL3*, *CCL4*, *CXCL3*, and *TNF*) and transcription factors (*FOS*, *JUN*, *ATF3*, and *NFKB* genes) associated with macrophage activation (Supplementary Table S5; refs. 21, 22).

Because of their known phenotypic similarities, we also re-clustered monocytes and DCs together. We identified clusters corresponding to classical monocytes (monocytes_*CD14*), nonclassical monocytes (monocytes_*CD16*), intermediary monocytes (monocytes_TNFSF13), conventional type 1 DCs, and conventional type 2 DCs (Supplementary Fig. S1E; Supplementary Table S6; refs. 23, 24).

In total, we identified 22 different immune cell types (Fig. 1D, Supplementary Fig. S1F; Supplementary Table S7). As expected, the frequency of some of these cell populations varied depending on their tissue of origin (Supplementary Fig. S2A and S2B). For example, B cells and myeloid cells were most prominent in lymph node biopsies and skin metastases, respectively. Importantly, biopsies from different tissues were evenly distributed across treatment outcomes (*χ*² test *P* value = 0.7; Supplementary Fig. S2C).

### NK Cell Abundance Is Associated with Intrinsic Resistance to ICB

As expected, activated, memory, and dysfunctional CD8^+^ T cells were more abundant in lesions from responders (R) than non-responders (NR; Fig. 1E). Surprisingly, NK cell abundance was significantly higher in OT lesions from NRs compared with responders whereas none of other immune cells showed the association with the response (Supplementary Fig. S3A). This observation was recapitulated in publicly available datasets, including: a scRNA-seq dataset of 48 biopsies collected before and after treatment (between days 22 and 867) from patients with metastatic melanoma exposed to αPD-1 therapy (16). Consistent with our data, a significant increase in NK cells was observed in OT lesions from NRs compared with responders [Supplementary Fig. S3B (left)]. Additionally, we leveraged a bulk RNA sequencing dataset from a metastatic melanoma cohort treated with αPD-1 therapy (25) and deconvolved the data using CIBERSORTx (26). Again, the relative abundance of NK cells in (± 3–4 weeks) OT samples was significantly higher in NRs [Supplementary Fig. S3B (middle)]. Notably, a similar association was observed in a scRNA-seq dataset of patients with breast cancer treated with αPD-1 and for which samples were collected 2 to 3 weeks OT (27). In this study, two populations of NK cells were identified: NK_Cyto_ (which compared with NK cells from OT metastatic melanoma lesions from NRs, expressing *GNLY*, *CXC3R1*, and *FCGR3A*) and NK_Rest_ (expressing *XCL1* and *XCL2*). Consistent with our findings in melanoma, the percentage of NK_Cyto_, but not NK_Rest_, was associated with a trend toward no clonotype T-cell expansion [Supplementary Fig. S3B (right)].

Three prominent NK cell subsets were recently described in healthy tissues: NK1, NK2, and NK3 (28). Most NK cells enriched in NR OT metastatic melanoma samples were related to the cytotoxic-like NK1 subset and were most similar to the NK effector state (group 3) and c7-NR4A3 population described in two recent pan-cancer studies (Fig. 1F; refs. 8, 29). In contrast, some of the NK cells in OT lesions from responders expressed *XCL1*, *XCL2* (∼50%, *P* = 0.12 and *P* = 0.00042, respectively, Wilcoxon test), and *IFNG* (∼25%, *P* = 0.34, Wilcoxon test; Fig. 1F) and were most similar to the NK2 cells (28) and NK stressed (group 1) and typical (group 2; ref. 29). This NK cell population exhibited immunoregulatory and cytokine-producing capacity (30). The ratios of chemokines/cytokines to the NK cytotoxicity gene score confirmed that the NK cells from the NRs exhibit a cytotoxic rather than a chemokine-producing phenotype (Fig. 1G).

Several NK cell populations have been correlated with immunotherapy resistance (6–8). However, the NK cells in our melanoma cohort differed significantly from those cells. Few cells expressed NCR3, and expression levels were very low, unlike the NKp30 population described by Kilian and colleagues (Fig. 1F; ref. 7). These cells were also clearly distinct from the tumor-associated CD69^+^IFNG^+^PRF1^–^ and the dysfunctional/low cytotoxic NR4A1^+^ CD158a (KIR2DL1)^+^ CD158e (KIR3DL1)^+^ NK cell populations described in the other studies (Fig. 1F).

Flow cytometry did not reveal differences in the percentage of total peripheral blood NK cells (PBNK), defined as CD19^−^ CD14^−^CD123^−^CD3^−^CD56^+^ cells (Supplementary Fig. S4A), across time points and ICB outcomes (Supplementary Fig. S4B; ref. 31). Notably, however, we observed that the fraction of GZMB^+^ and PRF1^+^ PBNKs decreased upon ICB exposure in NRs (but not in responders; Supplementary Fig. S4C). These data may indicate an active recruitment of these cells from the blood to NR, but not to responder, lesions during treatment.

These data suggest that an effector/cytotoxic NK cell population is enriched, possibly through active recruitment, in ICB-refractory lesions.

### Cytotoxic NK Cells Fail to Infiltrate the Tumor Bed in NR Lesions

**Figure 2.**
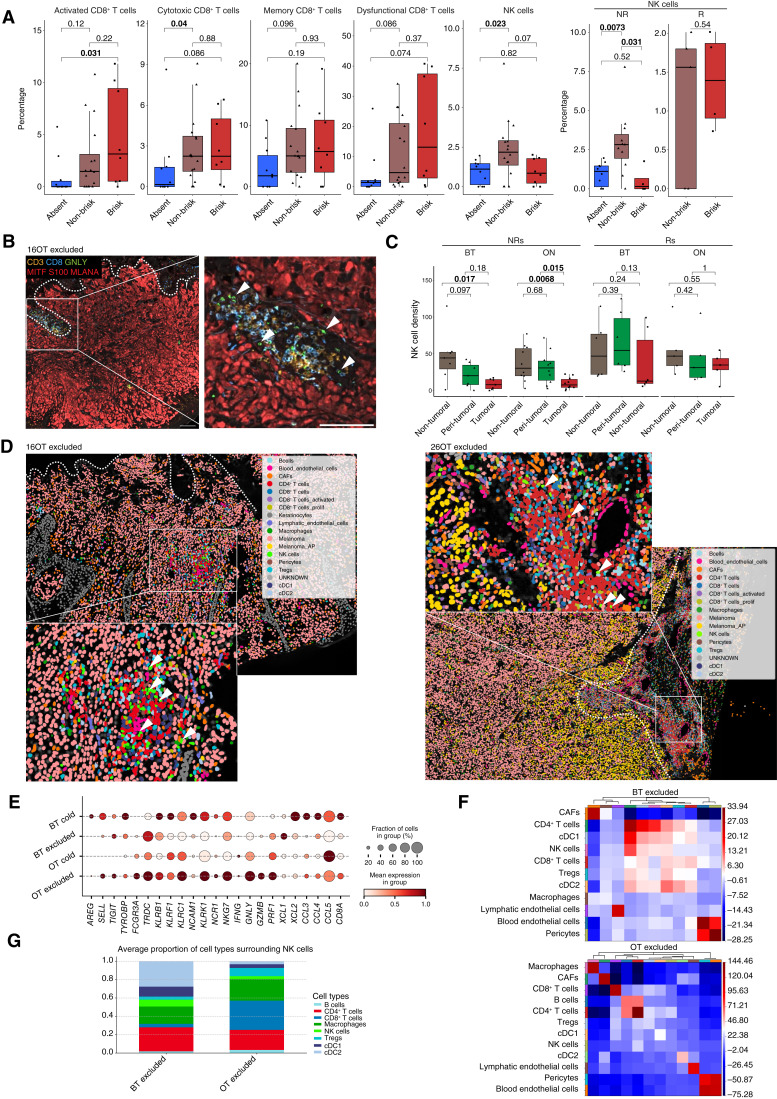
Spatial mapping of NK cells. **A,** Percentage of CD8^+^ T-cell subsets and NK cells of all immune cells plotted among three tumor-infiltrating lymphocytes: “absent” (cold), “non-brisk” (excluded), and “brisk” (hot) assessed by pathologist grouping all samples and splitting by response for the NK cells. Data analyzed by a two-sided Wilcoxon test and bold = *P* < 0.05. **B,** Representative multiplex immunofluorescence staining on lesions from an NR for whom NK cells were detected in the immune-infiltrating patch. Green = GNLY, red = melanoma, orange = CD3, and blue = CD8. Arrows point NK cells, and the dashed line represents tumor border. **C,** Density per square millimeter of NK cells compared between three different tumoral regions, response, and time point. Data analyzed by a two-sided Wilcoxon test and bold = *P* < 0.05. **D,** Spatial plots of all identified cells from two examples of immune-excluded tumors from skin metastasis biopsies. Arrows point toward NK cells, and the dashed line represents tumor border. **E,** Dotplot of expression of NK-related genes by NK cells plotted among immunophenotype and time point; BT cold, *n* = 1; BT excluded, *n* = 1; OT cold, *n* = 1; and OT excluded, *n* = 5; (**F**) Heatmaps of neighborhood enrichment of cell types/states identified using Xenium spatial transcriptomics for one BT excluded sample and five OT excluded samples. Cells for which abundance was <5, as well as cells annotated as either UNKNOWN or keratinocytes were removed from the neighborhood analyses. The scale represents the *z*-scores from a permutation test, indicating how frequently each pair of cell types was observed as neighbors compared with a randomly permuted spatial distribution. **G,** Stacked bar representing percentages of 20 immune cells surrounding NK cells, plotted between one BT excluded sample and five OT excluded samples. CAF, cancer-associated fibroblast; cDC1, conventional type 1 DCs; cDC2, conventional type 2 DCs; R, responder; Treg, regulatory T cell.

Not surprisingly, the abundance of the various CD8^+^ T subsets, and other immune cells, was significantly associated with a “brisk” infiltration pattern (Fig. 2A; Supplementary Fig. S5A). In contrast, NK cells were most numerous in biopsies exhibiting a “non-brisk” (immune-excluded) phenotype, which account for about 50% of all NR samples. Most remaining NR samples displayed an absent/desert phenotype.

To determine the spatial distribution of the NK cells, we performed multiplexed immunofluorescence in 17 NR samples and 11 responder samples, phenotyping individual cells with immune, melanoma, and stromal markers (*n* = 40; Supplementary Table S8). Guided by our scRNA-seq data, which showed that GNLY was only expressed in NK and cytotoxic CD8^+^ T cells, NK cells were identified as CD45^+^CD3^−^ and GNLY^+^ cells (Fig. 2B). Critically, NK cells were mainly localized in the non-tumoral and peri-tumoral areas in NR lesions, especially in OT samples. In contrast, NK cells were evenly distributed in responders at both time points (Fig. 2C). Similar observations were made for the CD8^+^ T cells (Supplementary Fig. S5B). Visual examination confirmed that NK cells are localized with CD8^+^ T cells at the tumor margin in NR lesions (Fig. 2C; Supplementary Fig. S5C). In responders, NK cells were part of a dense immune infiltrate surrounding and interspersed with melanoma cells [Supplementary Fig. S5D (top)] or were simply undetected [Supplementary Fig. S5D (bottom)].

To further explore the localization and phenotype of NK cells in NR lesions, we monitored the expression of 360 RNA markers (Supplementary Table S9) using high-plex *in situ* spatial transcriptomics on matched BT and OT biopsies (*n* = 4) and additional OT samples (*n* = 4). Leiden clustering identified various cell types/states, including NK cells [Supplementary Fig. S6A (left)]. We confirmed that most NK cells express high levels of *GNLY*, *PRF1*, and *GZMB* [Supplementary Fig. S6A (right)]. *XCL1*, *XCL2*, *IFNG*, *NCAM1*, *CD69*, and *CCL3* were expressed only in a minority of NK cells and at low levels, thus confirming the cytotoxic identity of most NK cells in NR samples. Most OT samples (five of six) harbored an immune-excluded phenotype [Fig. 2D; Supplementary Fig. S6B (left)]. In these samples, all NK cells were excluded from the tumor area and co-localized with CD4^+^ T cells, CD8^+^ T cells, DCs, and B cells either outside of the tumor parenchyma and/or surrounding blood vessels in regions lacking melanoma cells. One of the BT samples also harbored an immune-excluded phenotype [Supplementary Fig. S6C (left)] and one harbored a cold phenotype [Supplementary Fig. S6C (right)].

The expression of cytotoxic genes (e.g., *GNLY*, *GZMB*, and *PRF1*) was enriched in the excluded OT samples, and *XCL1* and *XCL2* were enriched in BT samples (Fig. 2E). Neighborhood analysis confirmed that NK cells co-localized with CD4^+^ T cells, CD8^+^ T cells, DCs, and B cells, most notably in the excluded OT samples (Fig. 2F; Supplementary Fig. S6D). Interestingly, when examining the identity of the 20 immune cells closest to the NK cells, we found CD4^+^ T cells and DCs in BT sample and CD8^+^ T cells in OT samples (Fig. 2G).

These data indicate that cytotoxic NK cells accumulate outside of the tumor area in ICB-exposed lesions from refractory patients exhibiting an immune-excluded phenotype.

### NK Cell Ablation Induces ICB Responsiveness in a Melanoma Mouse Model Exhibiting an Immune-Excluded Phenotype

**Figure 3.**
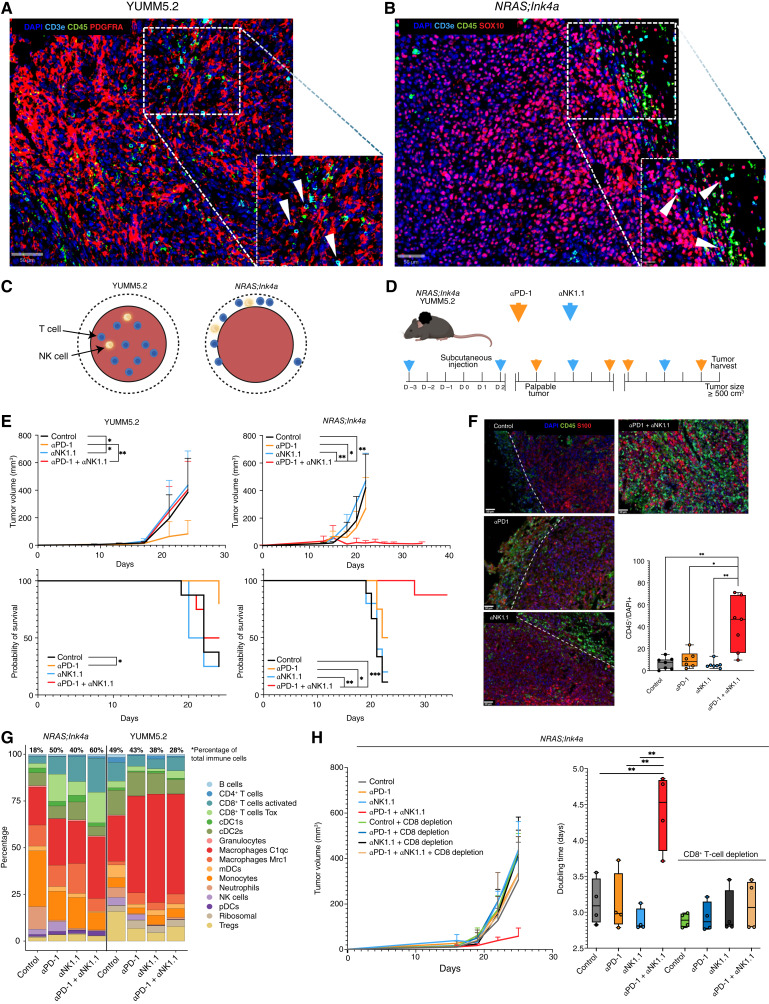
NK cells as modulators of ICB sensitivity in mice. **A,** Immunofluorescence of YUMM5.2 and (**B**) *NRAS;Ink4a* tumors. Tumors were stained for a melanoma marker (PDGFRa for YUMM5.2 and SOX10 for *NRAS;Ink4a*) and for CD45 and CD3ε to map the T-cell infiltration. Counterstaining was performed with DAPI. **C,** Schematic representation of NK and T-cell infiltration in the *NRAS;Ink4a* and YUMM5.2 tumors. **D,** Schematic representation of the therapeutic regimen used with YUMM5.2 and *NRAS;Ink4a* tumors. **E,** Growth curves of YUMM5.2 (top left) and *NRAS;Ink4a* tumors (bottom left) *in vivo* (*n* ≥ 5 per cohort (control, αPD-1, aNK1.1, and αPD-1 + aNK1.1). A Welch-corrected *t* test was performed on the doubling time of tumors upon exponential fitting (*, *P* < 0.05; **, *P* < 0.01). Kaplan–Meier curves representing progression-free survival of YUMM5.2 (top right) and *NRAS;Ink4a* tumors (bottom right) *in vivo* (*n* ≥ 5 per cohort. A log-rank Mantel–Cox test was used; *, *P* < 0.05; **, *P* < 0.01; ***, *P* < 0.001). **F,** Cryosection immunofluorescence showing the overall immune infiltrate of *NRAS;Ink4a* tumors across cohorts. Tumor cells were identified by S100 and immune cells by CD45. Counterstaining was performed with DAPI. The dashed line marks the separation between tumor and immune areas. Quantification of overall immune infiltration in *NRAS;Ink4a* tumors (number of CD45^+^ of all DAPI-positive cells, with *n* ≥ 6 per cohort and data analyzed with a Welch-corrected *t* test; *, *P* < 0.05; **, *P* < 0.01). **G,** Stacked bar plot showing proportions of immune cell types from the *NRAS;Ink4a* and YUMM5.2 tumors across conditions based on the scRNA-seq experiment. For each condition, at least four mice were pooled (see “Methods”). **H,** Growth curves (left) and doubling time calculation (right) of *NRAS;Ink4a* tumors *in vivo* treated with αPD-1 and/or aNK1.1 in the presence/absence of CD8^+^ T-cell depletion (*n* = 4 per cohort and a Welch-corrected *t* test was performed on the doubling time of tumors upon exponential fitting; **, *P* < 0.01). cDC1, conventional type 1 DCs; cDC2, conventional type 2 DCs; mDC, migratory DCs, Treg, regulatory T cell. (**C** and **D,** Created with BioRender.com.)

To determine whether NK cells play a causative role in ICB resistance in lesions with an immune-excluded phenotype, we treated mice bearing either the immune-excluded *NRAS;Ink4a* tumors (32) or the immune-infiltrated YUMM5.2 tumors (Fig. 3A–C; Supplementary Fig. S7A; ref. 33) with αPD-1 and/or anti-NK1.1 (aNK1.1) antibodies to deplete NK cells (Fig. 3D). Although the administration of αPD-1 alone reduced the growth of YUMM5.2 allograft melanoma lesions, this treatment had only a marginal impact on *NRAS;Ink4a* tumors (Fig. 3E). NK cell depletion in the immune-infiltrated YUMM5.2 model abrogated the sensitivity to ICB, whereas it drastically sensitized the *NRAS;Ink4a* model to αPD-1 therapy (Fig. 3E). Histopathologic analyses revealed that treatment with either aNK1.1 or αPD-1 alone did not affect integrity of the tumor compartment in this model [Supplementary Fig. S7B (bottom row)]. Although αPD-1 led to a moderate (although not significant) increase in CD45^+^ tumor infiltration, aNK1.1 did not cause any change in the immune infiltration pattern [Fig. 3F; Supplementary Fig. S7C (right)]. The combination of these antibodies, however, resulted in a massive increase in immune cell infiltration and tumor destruction. This was in sharp contrast to the observations in the YUMM5.2 [Supplementary Fig. S7C (left)] model and in YUMM1.7, another immune-infiltrated model (Supplementary Fig. S8A and S8B). Thus, NK cells enhance ICB efficacy in melanoma with an immune-infiltrated phenotype but hinder efficacy in lesions with an immune-excluded phenotype. Remarkably, in the accompanying article, similar findings are reported in the immune-excluded model of colon adenocarcinoma (MC38; refs. 34, 35)

### NK Cells Compromise CD8^+^ T-cell Antitumor Function in Immune-Excluded Melanoma

To gain insights into the mechanisms underlying NK-mediated resistance to ICB, we analyzed the cellular composition of *NRAS;Ink4a* and YUMM5.2 melanomas across cohorts by scRNA-seq (Supplementary Fig. S9A and S9B; Supplementary Table S10). Similar to the observations in human biopsies from NR patients, αPD-1 caused an increase in NK cell abundance in *NRAS;Ink4a* lesions. In contrast, the same treatment led to a decrease in NK cells in YUMM5.2 tumors. CD8^+^ T cells increased in *NRAS;Ink4a* lesions upon NK cell depletion, and this was exacerbated upon αPD-1 and aNK1.1 combined exposure (Fig. 3G; Supplementary Fig. S9C). This observation suggests that in the *NRAS;Ink4a* immune-excluded tumors, NK cells compromise the expansion of the CD8^+^ T-cell compartment. Importantly, NK cell depletion also led to the accumulation of CD8^+^ T cells in the MC38 model (see the accompanying article; ref. 34).

In the *NRAS;Ink4a* model, CD8^+^ T cells clustered outside of the tumor area (Supplementary Fig. S9C and S9D). Exposure to aNK1.1 did not alter this pattern, and treatment with αPD-1 slightly increased CD8^+^ T-cell infiltration. In contrast, combined treatment resulted in a dramatic recruitment of CD8^+^ T cells within the tumor bed. These results establish a role for NK cells in the maintenance of the immune-excluded phenotype in lesions exposed to ICB.

We next transplanted *NRAS;Ink4a* cells in mice lacking CD8^+^ T cells (Supplementary Fig. S9E). Although NK cell depletion combined with αPD-1 treatment induced a drastic antitumor response in control mice, this antitumor effect was completely abrogated in CD8^+^ T cell–deficient mice (Fig. 3H). This experiment provides direct evidence for a functional interaction between NK and CD8^+^ T cells in immune-excluded lesions.

### Targeting CX3CR1-Dependent NK Cell Recruitment Restores Immunotherapy Effectiveness

**Figure 4.**
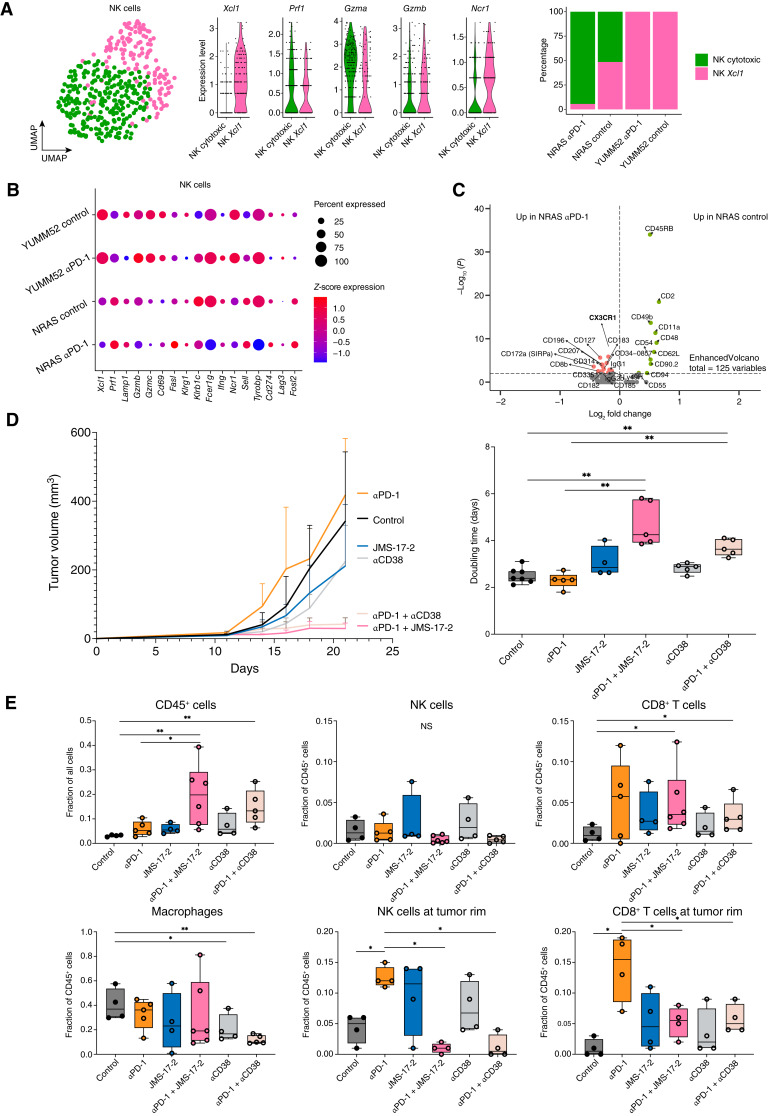
Pharmacologic targeting of NK cells. **A,** Uniform Manifold Approximation and Projection (UMAP) of re-clustered NK cells from the *NRAS;Ink4a* and YUMM5.2 tumors (left), selected genes expression between NK cell clusters (middle), and proportions of NK cells from the *NRAS;Ink4a* and YUMM5.2 tumors compared between control- and αPD-1–treated condition (right) based on the scRNA-seq experiment. **B,** Dot plot of the expression of selected genes between *NRAS;Ink4a* and YUMM5.2 tumors in NK cells only based on the scRNA-seq experiment. **C,** Volcano plot representing differentially expressed proteins in NK cells from the *NRAS;Ink4a* tumor between control and αPD-1 treatment. Significantly expressed proteins were considered based on the adjusted *P* value > 10e–3 based on the CITE-seq experiment. **D,** Growth curves (left) and doubling time calculation (right) of *NRAS;Ink4a* tumors *in vivo* treated with αPD-1, JMS-17-2, and CD38 (*n* ≥ 4 per cohort and a Welch-corrected *t* test was performed on the doubling time of tumors upon exponential fitting; **, *P* < 0.01). **E,** Quantification of immune cells (CD45^+^), NK cells (CD45^+^NKP46^+^), CD8^+^ T cells (CD45^+^CD3*ε*^+^CD8*α*^+^), and macrophages (CD45^+^F4/80^+^) in multiplexed immunofluorescence images (each dot corresponds to a single tumor; data analyzed with a Welch-corrected *t* test; *, *P* < 0.05; **, *P* < 0.01). Tumor rim corresponds to an area ≤0.2 mm^2^ in proximity to the tumor border.

We identified two main NK cell subclusters in melanoma mouse lesions exposed to ICB [Fig. 4A (left)]. One cluster, enriched in YUMM5.2 and characterized by high expression levels of *Xcl1*, closely resembled the NK cells detected in the human responder biopsies. The second cluster (enriched in the *NRAS;Ink4a* model) expressed high levels of *Prf1* and *Gzma*, resembled the cytotoxic NK cells identified in NR lesions, and drastically increased upon αPD-1 therapy (Fig. 4A and B).

Further phenotyping of the NK cell population of the *NRAS;Ink4a* model using Cellular Indexing of Transcriptomes and Epitopes by Sequencing (CITE-seq; cluster 11; Supplementary Fig. S10A; Supplementary Table S11) revealed that αPD-1 caused an increase in CX3CR1 protein expression and a decrease in CD62L, integrins (CD49b and CD11a), CD54 (*Icam1*), and CD90.2 (*Thy1*; Fig. 4C), suggesting that αPD-1 promotes the active recruitment of NK cells to these lesions. We also observed an upregulation of CD355 (Nkp46) and CXCR1, indicating that the NK cells mature under ICB therapy. Likewise, αPD-1 induced accumulation of mature (CD62L^–^) and cytotoxic (CD69^+^GZMB^+^) NK cells in the MC38 colon cancer model (Supplementary Fig. S10B).

We treated *NRAS;Ink4a*-bearing mice with the chemical inhibitor of CX3CR1, JMS-17-2 (36). Although JMS-17-2 did not significantly alter the *in vivo* growth kinetics of these tumors, the combination with αPD-1 led to a dramatic inhibition of tumor growth (Fig. 4D; Supplementary Fig. S10C). Consistently, we observed a significant decrease in NK cell abundance at the tumor rim and an increased infiltration of CD45^+^ immune cells (including CD8^+^ T cells) upon exposure to the αPD-1 and JMS-17-2 combination (Fig. 4E; Supplementary Fig. S10D). These data indicate that the recruitment of NK cells induced by ICB is dependent on CX3CR1 and highlight the key contribution of this recruitment to immune exclusion and ICB unresponsiveness.

### A Clinically Compatible Approach to Mitigate Therapy Resistance in Tumors with an Immune-Excluded Phenotype

Daratumumab, a monoclonal antibody directed against CD38, is approved for the treatment of patients with multiple myeloma (37). Long-term treatment with daratumumab causes an increased risk of infection due to the depletion of PBNKs (37). Interestingly, CD38 targeting did not significantly alter the *in vivo* growth of *NRAS;Ink4a* lesions, but the combination of anti-CD38 (αCD38)/αPD-1 significantly reduced tumor growth (Fig. 4D). This was accompanied by an induction of intratumoral necrosis (Supplementary Fig. S10C), a significant decrease in NK cell abundance at the tumor rim, and an increase in CD8^+^ T-cell tumor infiltration (Fig. 4E; Supplementary Fig. S10D). Notably, aCD38 exposure also decreased intra-tumoral macrophage abundance (Supplementary Fig. S10D). This may be caused by the depletion of a population of macrophages expressing CD38 present in these lesions (cluster 2, Supplementary Fig. S10A; Supplementary Table S11) and/or secondary to the increase in CD8^+^ T-cell tumor abundance. Nevertheless, these findings present an approach to address ICB therapy resistance in tumors with an immune-excluded phenotype that can be rapidly implemented in clinical settings.

## Discussion

This study characterizes the immune landscape of treatment-naïve melanoma in more than 20 patients and its response to immunotherapy. The resulting dataset, accessible via a user-friendly online application, offers a valuable resource for advancing melanoma biology.

Using these data, we unexpectedly observed an increase in cytotoxic NK cell abundance in OT lesions from ICB-refractory patients and replicated this finding in independent melanoma and breast cancer cohorts. In keeping with this, three recent studies described an association between NK cells and lack of response to immunotherapy in different cancer types (6–8). However, gene expression analyses indicated that the NK populations described in these studies are distinct from the NK cells we identified in our OT NR patients with metastatic melanoma.

Given that the NK cell population we describe exhibits a mature and cytotoxic phenotype, our observation starkly contrasts with previous reports in which the abundance of cytotoxic NK cells was linked to response to αPD-1 therapy (38). Spatial analysis revealed that increased NK cell abundance was confined to peri-tumoral regions, near CD8^+^ T cells and DCs, potentially explaining the discrepancy. Thus, comparing NK cell abundance across samples and studies will require consideration of the diversity in NK cell phenotypes and their spatial distribution. Nevertheless, our study indicates that the presence of cytotoxic NK cells at the tumor border of NR lesions at an early OT time point may be a predictive biomarker.

Critically, we provide direct evidence that the increase in cytotoxic NK cells in the peri-tumoral area upon ICB exposure is causatively involved in promoting resistance to ICB. Improved ICB response was indeed observed after NK cell depletion in immune-excluded melanoma and colon cancer models. Conversely, NK cell depletion impaired ICB response in immune-infiltrated melanomas (YUMM5.2 and YUMM1.7), indicating context-dependent effects of NK cells on ICB efficacy.

In immune-excluded melanoma, depletion of NK cells led to an increase in CD8^+^ T cells, a phenotype that was exacerbated upon αPD-1 exposure. These data indicate that, in this tumor context, ICB therapy induces NK cell recruitment and/or promotes their maturation/cytotoxicity in the “wrong” compartment, leading to a decrease in CD8^+^ T-cell abundance and dampened immune cell infiltration. Mechanistically, the recruitment of NK cells to these inflamed areas relies on a CX3CR1-dependent pathway as pharmacologic targeting of this pathway reverts immune exclusion and enhances the sensitivity to αPD-1 treatment in a representative mouse model. However, we cannot rule out that by using CX3CR1 antagonist, other immune cell types can be targeted. Nevertheless, in this scenario, CD8^+^ T cells are critical for ICB response as genetic ablation of CD8^+^ T cells is sufficient to abrogate the antitumor response induced by the aNK1.1/αPD-1 combination treatment. The accompanying article supports a direct functional interaction between NK and CD8^+^ T cells and proposes that NK cells act by preventing the differentiation of CD8^+^ T cells toward a highly cytotoxic/effector cell population (34).

These findings have key therapeutic implications as immune-exclusion is common in ICB-resistant patients. By revealing the role of cytotoxic NK cells in maintaining this immunophenotype, new treatment strategies will emerge. We present preclinical evidence that targeting NK cells, using aCD38 for instance, merits clinical evaluation. Conversely, in immune-infiltrated melanoma, blocking NK cell inhibition (39) could enhance ICB response. As NK cell–based therapies gain traction, our data also highlight the importance of considering their dual role in the tumor context.

## Methods

### Patient Samples

Tumor biopsies were collected as part of a non-interventional prospective study investigating transcriptomic changes upon immune checkpoint inhibition (Prospective Serial biopsy collection before and during immune checkpoint inhibitor therapy in patients with malignant melanoma; SPECIAL). Patient recruitment and sample collection were previously described (13). In brief, metastatic lesions of treatment-naïve patients with advanced (stage III and stage IV) cutaneous melanoma treated with αPD-1–based ICB (either in neoadjuvant or inoperable setting) were biopsied right before the administration of the first and second ICB cycle. Demographic, clinical, histopathologic, and genetic information were collected at baseline. Patients with unresectable disease were stratified as responders (complete remission and partial remission) and NRs (stable disease and progressive disease) based on RECIST v1.1 best overall response (for clarity, it was assessed after 12 weeks, not at the time of the second biopsy), whereas patients treated with curative intent were stratified according to pathologic response assessment at tumor resection. Written informed consent was obtained from all patients. All study procedures were in accordance with the principles of the Declaration of Helsinki and applicable Belgian law and regulations and approved by the UZ Leuven Medical Ethical Committee (S62275 and S62927).

### scRNA-seq on Human Samples

Methods for tumor dissociation, library construction, and sequencing and scRNA-seq data acquisition and analysis were described previously (13). Immune cells were identified using a gene set score of >0.1 for the immune score from Jerby-Arnon and colleagues (14), in addition to an inferred mean copy-number variation score <0.1. Next, the SCTransform was applied regressing out mitochondrial read percentage and cell cycle scores, and the data integration (by samples) was performed using the R package Harmony v. 1.0 (40). The main immune cell types (*n* = 17) were identified after the first round of unsupervised Louvain clustering and marker gene calling. Next, all CD8^+^ T-cell clusters were re-clustered together for deeper characterization. Myeloid and pDC clusters were further re-clustered, followed by re-clustering of macrophages separately and DCs together with monocytes. The marker genes of each identified cluster were calculated using the FindAllMarkers function in Seurat (Wilcoxon rank-sum test).

Stromal components (cancer-associated fibroblasts and endothelial cells) were identified based on gene signatures from Jerby-Arnon and colleagues (14). Once all the immune cell types were identified and annotated, their relative abundance to all immune cells in each sample was compared between responders and NRs and time point using the Wilcoxon rank-sum test when comparing between the response status and the paired Wilcoxon rank-sum test when comparing between time points.

### Validation of NK Cells in an Independent scRNA-seq Dataset

The transcript per million–normalized Sade-Feldman and colleagues dataset was downloaded from the Gene Expression Omnibus portal (accession number GSE120575; ref. 16). Importantly, samples in this study were recruited from a mixed cohort which included patients treated with αCTLA4 in monotherapy. Therefore, we restricted our validation analyses to patients treated with only αPD-1 and in combination with αCTLA4 treatment. Cells with >1,000 and <7,500 expressed genes were selected for further analysis. NK cells in this independent dataset were identified within G8 (cytotoxicity) cluster based on an AUCell score of the differentially expressed genes in the NK cell cluster in our dataset compared with the CD8^+^ T cells (Supplementary Table S3). Additionally, cells positive for CD8A were excluded. Cells positive for this score were considered as NK cells. Then the fraction of NK cells of all immune cells was compared between responders and NRs and time point using the Wilcoxon rank-sum test.

CIBERSORTx was used to infer tumor microenvironment components and main immune cells using our human scRNA-seq dataset as a reference: B cells, endothelial cells, macrophages, CD8^+^ T cells, T regulatory cells, malignant cells, cancer-associated fibroblasts, DCs, CD4^+^ T cells, and NK cells from the Riaz and colleagues (25) bulk RNA sequencing dataset (accession number GSE91061). Count matrices of our scRNA-seq data were subsampled for 15,000 cells and used to generate cell type expression signatures with default settings except for the minimal expression parameter, which was set to 0. The tumor microenvironment cell types were imputed with default parameters with batch correction mode.

CIBERSORTx was run with rmbatchSmode = TRUE, nsampling = 30, and QN = FALSE. The fraction of deconvolved NK cells in the Riaz and colleagues cohort was compared between responders and NRs with ICB and time points using the Wilcoxon rank-sum test.

### Multiplex Imaging and Digital Pathology

Multiplex iterative labeling by antibody neodeposition platforms were carried out as previously described (13) on formalin-fixed, paraffin-embedded (FFPE) tissue biopsies: 17 NR samples (BT, *n* = 7; OT, *n* = 10) and 11 responder samples (BT, *n* = 6; OT, *n* = 5; Supplementary Table S1B). Multiplex iterative labeling by antibody neodeposition entails multiple rounds of indirect immunofluorescent staining using unconjugated antibodies, followed by imaging and antibody removal via detergent and a reducing agent. Image analysis was performed as previously described (41). Briefly, stains were visually evaluated for quality by an experienced pathologist. Flat-field correction was performed using a custom implementation of a publicly described methodology (42). Consecutive staining rounds were registered using a publicly available algorithm (43). Tissue autofluorescence was subtracted using a baseline image stained only with a secondary antibody. Nuclear cell segmentation was performed using a fine-tuned version of StarDist (44). Phenotypic identification was done based on prior knowledge using a two-tiered approach combining different clustering methods with functional analysis. Finally, the tissue was digitally reconstructed to perform spatial analysis. Antibodies used in this experiment are listed in Supplementary Table S8. NK cells defined as CD3^−^GNLY^+^ cells were counted per square millimeter unless otherwise specified.

### Spatial Transcriptomics

Spatial transcriptomic analysis was performed using the 10x Xenium Analyzer v1.9, which allows for detection and subsequent in-depth spatial analysis of hundreds of RNA molecules. Our panel is composed of the 10x predesigned “Human Skin” panel (282 genes) and a custom add-on panel (80 self-selected genes). The selection of samples and area of interest for analysis was carried out in collaboration with the involved, experienced pathologist (F.B.) and guided by available hematoxylin and eosin sections. The hematoxylin and eosin sections together with DV200 index assessment of obtained trim sections served as quality control before proceeding to the actual analysis. Tissue sections were processed according to manufacturer’s guidelines. Briefly, after rehydration, tissue sections of 5-μm thickness were cut from FFPE tissue blocks, followed by passive drying for 30 minutes at room temperature, incubation for 3 hours at 42°C, and dry storage at room temperature. On the day of the actual processing, slides were incubated for 30 minutes at 60°C, followed by deparaffinization and decrosslinking. Immediately afterward, overnight probe hybridization was set in, in which the DNA probe mixture was added to the tissue to hybridize with the target RNA molecules. The DNA probes hold two terminal RNA target sites which, after hybridization with complementary target RNA, give the DNA probe a circular shape, releasing a central site with gene-specific barcode and primer-binding region. On the second day, ligation takes place in which, after rinsing unbound probes, a ligase reaction seals the junction between probes and target RNA. Subsequently, enzymatic amplification of the gene-specific barcodes is conducted. Finally, after 4′,6-diamidino-2-phenylindole dihydrochloride (DAPI) staining, imaging and decoding of fluorescence signals in Xenium Analyzer was performed.

The Xenium data, containing a DAPI-stained image and a table with the corresponding transcript locations per sample, were processed using the SPArrOW pipeline (bioRxiv 2024.07.04.601829). The images were cropped to remove substantial artefact-empty and artefact-rich regions. All transcripts located within the segmentation mask of a nucleus were summed per gene, resulting in a cell-by-gene count matrix. Next, the data from all samples were merged. Genes that exhibited expression in fewer than five cells and cells with a total count lower than 10 were filtered out. To normalize the counts, the size of the nucleus was used as a scaling factor before applying a log transformation. Next, the ensuing counts were scaled and used as input for further cell type/state annotation using SPArrOW, Scanpy (45), and Squidpy (46). All the cells were then clustered using the sparrow function sp.tb.cluster, with arguments neighbors = 50, pcs = 40, and cluster_resolution = 4. The cells were annotated based on the marker genes, and NK cells were further validated with the NK cell gene signature derived from the scRNA-seq data. Clusters without clear identity were named as “UKNOWN”. Finally, the neighborhood enrichment was calculated using the sq.gr.nhood_enrichment squidpy function, after removing cells for which abundance was <5, as well as cells annotated as either UNKNOWN or keratinocytes.

### Flow Cytometry of Human Peripheral Blood Mononuclear Cells

Patient blood samples were obtained at the time of tumor biopsy in 3× 10 mL EDTA tubes: 28 NR samples (BT, *n* = 13; OT, *n* = 15) and 23 responder samples (BT, *n* = 12; OT, *n* = 11). In addition, we collected blood from age- and sex-matched controls (*n* = 10; Supplementary Table S1C). Peripheral blood mononuclear cells were isolated using Ficoll-Paque density gradient centrifugation. First, blood was diluted 1:1 in sterile Dulbecco’s PBS (DPBS; Thermo Fisher Scientific, cat. #14-200-075). This mixture was carefully layered on top of 10 mL of sterile Ficoll-Paque Plus (Sigma-Aldrich, cat. #GE17-1440-02) and centrifuged for 30 minutes at 400 × *g* at room temperature. Then the diluted plasma layer on top was pipetted off and the interface layer containing the peripheral blood mononuclear cells was carefully collected. The cells were resuspended in DPBS and washed twice before cryopreservation in 10% DMSO (Sigma-Aldrich, cat. #472301) in FBS (VWR, cat. #S1810-500) at −80°C.

For flow cytometry, the cells were thawed (0.5 × 10^6^ per staining), incubated with human FcR block (5 μL/sample, Miltenyi Biotec), and stained with monoclonal antibodies (list of human antibodies is shown in Supplementary Table S12). Dead cells were excluded using Zombie Aqua 516 (1:1,000, BioLegend) or Fixable Viability Stain 620 (1:25,000, BD Biosciences). Flow cytometric analysis was performed on a BD LSRFortessa X-20 with DIVA software. Results were analyzed with FlowJo (LLC, V10).

### Mouse Experiments

#### Cell Lines

The *NRAS;Ink4a* cell line was generated in the lab (32), and YUMM5.2 was kindly provided by professor Colinda Scheele (VIB KU Leuven CCB). These cell lines were cultured in DMEM 4.5 g/L glucose (Gibco, Thermo Fisher Scientific) supplemented with 5 mL minimum essential medium nonessential amino acids (Gibco, Thermo Fisher Scientific), 5 mL 10,000 U/mL penicillin–streptomycin (Gibco, Thermo Fisher Scientific), and 10% FBS. Cells were passaged and detached using DPBS (1X; Gibco, Thermo Fisher Scientific) and 0.05% trypsin–EDTA (1X; Gibco, Thermo Fisher Scientific). Cells were maintained in culture no more than 4 weeks in a row and never beyond passage 30. Our cell lines are routinely tested (every 2 weeks) for *Mycoplasma* detection according to established protocol (47).

#### Mice

Male and female C57BL/6 mice were purchased from KU Leuven Mouse Facility and housed at 22°C ± 2°C, 55% ± 10% relative humidity, and with 12 hours day/light cycles in mouse facilities at the KU Leuven Mouse Facility (Leuven, Belgium). *In vivo* studies were performed after approval from the KU Leuven Ethical Committee for Animal Experimentation (ECD numbers 188/2022 and 205/2024).

To assess the effects of NK cell depletion on ICB response in murine models, 2.5 × 10^5^*NRAS;Ink4a* cells were subcutaneously injected into 8- to 12-week-old female C57BL/6 mice, whereas 2.5 × 10^5^ YUMM5.2 cells were subcutaneously injected into 8- to 12-week-old male C57BL/6 mice (cohort size is reported in the Fig. 4 legend). Tumor volume was monitored every other day and calculated using the formula V = (width  × length^2^)/2. As soon as tumors reached a volume of ∼20 mm^3^ (indicatively corresponding to a palpable 3 × 3 mm mass), mice were administered either αPD-1 (clone RMP1-14) or IgG2a control isotype (Bio X Cell; 200 μg in 200 μL PBS, twice per week, intra-peritoneal injection). To perform NK cell depletion, mice were administered aNK1.1-depleting antibody (Bio X Cell; 100 μg in 200 μL PBS; clone PK136) 3 days before tumor injection and every 5 days after tumor injection. Mice were sacrificed (by cervical dislocation) in all the cohorts once the tumor volume in the control cohort reached ∼500 mm^3^. We decided to uniquely maintain the αPD-1 + anti-NK1.1 cohorts longer in the *NRAS;Ink4a* model to appreciate the extent of prognosis improvement upon treatment. Tumors from the *NRAS;Ink4a* model were then included in cryosections as described below.

To assess the effect of either aCD38 or JMS-17-2 on ICB response in the *NRAS;Ink4a* model, 2.5 × 10^5^*NRAS;Ink4a* cells were subcutaneously injected into 8- to 12-week-old female C57BL/6 mice (cohort size is reported in the Fig. 4 legend). Tumor volume was monitored every other day and calculated using the formula V = (width  × length^2^)/2. As soon as tumors reached a volume of ∼20 mm^3^ (indicatively corresponding to a palpable 3 × 3 mm mass), drug administration was started according to the following regimen: (i) IgG2a control isotype 200 μg in 200 μL PBS, twice per week by intraperitoneal injection; (ii) αPD-1 (clone RMP1-14; Bio X Cell) 200 μg in 200 μL PBS, twice per week by intraperitoneal injection; (iii) aCD38 (clone NIMR5, Bio X Cell) 10 mg/kg in 100 μL PBS, twice a week by intraperitoneal injection; and (iv) JMS-17-2 (MedChemExpress, cat. #HY-123918) 10 mg/kg in 100 μL PBS, five times per week by intraperitoneal injection. Mice were sacrificed (by cervical dislocation) in all the cohorts once the tumor volume in the control cohort reached ∼500 mm^3^.

### scRNA-seq and Data Processing

Mice were housed and transplanted as described above (mouse experiments section; *n* = 4 for control and aNK1.1 cohorts for both *NRAS;Ink4a* and YUMM5.2; *n* = 5 for αPD-1 and αPD-1 + aNK1.1 cohorts for both *NRAS;Ink4a* and YUMM5.2). Tumor volume was monitored every other day and calculated using the formula V = (width  ×  length^2^)/2. As soon as tumors reached a volume of ∼20 mm^3^ (indicatively corresponding to a palpable 3 × 3 mm mass), mice were administered either αPD-1 (clone RMP1-14) or IgG2a control isotype (Bio X Cell; 200 μg in 200 μL PBS, twice per week). To perform NK cell depletion, mice were administered aNK1.1-depleting antibody (Bio X Cell; 100 μg in 200 μL PBS; clone PK136) 3 days before tumor injection and every 5 days after tumor injection. Mice were sacrificed (by cervical dislocation) in all the cohorts once the tumor volume in the control cohort reached ∼500 mm^3^. Upon sacrifice, tumors were collected and divided in two. A part of the tumor was processed for multiplexed immunofluorescence (see below), whereas the rest was freshly chopped and then digested with collagenase P (2 mg/mL) and DNase I (0.2 mg/mL) for 30 minutes at 37°C. Tumors from the same cohorts were pooled together at this stage. After digestion, digestion medium was inactivated with 100% FBS and tumor cells were filtered with 70-μm strainer. Tumor cells were centrifuged (5 minutes at 1,300 rpm and 4°C) and red blood cells were lysed in 2 mL NH_4_Cl (0.15 mmol/L), NaHCO3 (0.01 mmol/L), and EDTA (0.001 mmol/L) for 5 minutes on ice. Lysis buffer was then inactivated by topping the volume up to 50 mL with ice-cold PBS. The cells were then centrifuged (5 minutes at 1,300 rpm and 4°C) and resuspended in 2 mL of ice-cold PBS. The cells were counted with trypan blue; as the protocol led to viability >75%, no enrichment for live cells was performed. The cells were resuspended in PBS + 0.04% BSA at an estimated final concentration of 1,000 cells/μL and loaded on a Chromium GemCode Single Cell Instrument (10x Genomics) to generate single-cell gel beads-in-emulsion (GEM). The scRNA-seq libraries were prepared using the GemCode Single Cell 5′ Gel Bead and Library kit, version Next GEM 2 according to the manufacturer’s instructions (10x Genomics, CG000331). The cDNA content of pre-fragmentation and post–sample index PCR samples was analyzed using the Qubit Flex Fluorometer (Life Technologies). The fragment size of every library was analyzed using the 5200 Fragment Analyzer (Agilent). Sequencing libraries were loaded on an Illumina NovaSeq flow cell at the VIB Nucleomics core with sequencing settings according to the recommendations of 10x Genomics (paired-end reads 28-10-10-90, 1% PhiX).

The raw sequencing reads were processed by CellRanger (v. 7.2.0, 10x Genomics) with the mouse reference genome v. mm10.

Raw count matrices were analyzed using the R package Seurat v. 5.0.3 (48). The matrices were filtered by keeping cell barcodes with >500 expressed genes, <6,000 expressed genes, and <10% of reads mapping to mitochondrial reads. scDblFinder (49) was used to filter out doublets. SCTransform was applied by regressing out mitochondrial read percentage and cell cycle scores. Subsequently, the data integration was performed using the scVIIntegration function in Seurat.

Immune and malignant signatures were acquired from Jerby-Arnon and colleagues (14) and Tirosh and colleagues (50) to calculate gene set scores using the R package AUCell v. 1.6.1(51). The immune compartment was identified by using the immune signature cutoff suggested by the AUCell package. Next, the immune compartment was subjected to SCTransform and scVIIntegration and re-clustered with parameters dims = 1:30 and resolution = 0.3. Cluster assignment to immune cell type was identified based on SingleR annotations and marker genes. Similarly, NK cells were re-clustered with dims = 1:10 and resolution = 0.5.

### CITE Sequencing and Data Processing

When preparing the cells for scRNA-seq, a part of the cell suspension from the control and αPD-1 cohorts in *NRAS;Ink4a* tumors was further processed for CITE-seq. To this aim, CD45^+^ immune cells were initially enriched with the EasySep Mouse CD45 Positive Selection Kit (https://cdn.stemcell.com/media/files/pis/10000005216-PIS_02.pdf; STEMCELL Technologies). Briefly, the cells were initially resuspended in ice-cold PBS at the final concentration of 10^8^ cells/mL and then incubated with the selection antibody cocktail (100 μL/mL of sample) for 5 minutes at room temperature in a 5 mL (12 × 75 mm) polystyrene round-bottom tube. Afterward, the cells were incubated with RapidSpheres for 3 minutes at room temperature and then the volume was topped up to 2.5 mL with PBS. The cell suspension was mixed by pipetting and then the tube was placed in the EasySep Magnet for 5 minutes. The supernatant was discarded and the last two steps were repeated. For CITE-seq labeling, 2 million cells were counted, isolated, and spun down. The cell pellet was resuspended and incubated for 30 minutes on ice with 50 µL of staining mix in PBS containing 0.04% BSA, TruStain FcX Block (BioLegend, cat. #101320), the mouse cell surface protein antibody panel containing 184 oligo-conjugated antibodies, and nine TotalSeq-C isotype controls (TotalSeq-C, BioLegend; Supplementary Table S13). The cells were washed 3× with PBS + 0.04% BSA. Single-cell suspensions were resuspended at an estimated final concentration of 1,000 cells/μL and loaded on a Chromium GemCode Single Cell Instrument (10x Genomics) to generate single-cell GEM. The scRNA-seq and Feature Barcoding libraries were prepared using the GemCode Single Cell 5′ Gel Bead and Library kit, version Next GEM 2 according to the manufacturer’s instructions (10x Genomics, CG000330). The cDNA content of pre-fragmentation and post–sample index PCR samples was analyzed using the Qubit Flex Fluorometer (Life Technologies). The fragment size of every library was analyzed using the 5200 Fragment Analyzer (Agilent). Sequencing libraries were loaded on an Illumina NovaSeq flow cell at the VIB Nucleomics core with sequencing settings according to the recommendations of 10x Genomics (paired-end reads 28-10-10-90, 1% PhiX).

The raw sequencing reads were processed by CellRanger (v. 7.2.0, 10x Genomics) with the mouse reference genome v. mm10.

Subsequently the data were processed in the same manner as for the scRNA-seq data (however without scVIIntegration) with parameters for immune cells re-clustering dims = 1:10 and resolution = 10. The NK cells were subset and differential protein expression was performed using the FindMarkers function in Seurat. Volcano plot was plotted using the EnhancedVolcano package in R (https://github.com/kevinblighe/EnhancedVolcano).

### Immunofluorescence on Cryosections

To assess the immune cell infiltration in *NRAS;Ink4a* tumors across multiple cohorts, tumors were resected, rinsed in ice-cold DPBS, fixed in 4% formaldehyde for 2 hours, washed in DPBS, and placed in 30% sucrose in DPBS overnight. Next, tissues were embedded in Tissue-Tek-O.C.T. Compound (Sakura-Finetek, #4583) and stored at −80°C. Sections of 10 μm were cut using the Thermo Fisher Scientific CryoStar NX70 Cryostat. For immunofluorescence, tissue sections were air dried, rinsed in DPBS, and fixed for 10 minutes in 4% paraformaldehyde. They were washed in DPBS for 5 minutes, permeabilized in 1% Triton X-100 in DPBS for 10 minutes, and washed in DPBS for 5 minutes again. Next, the sections were incubated in blocking buffer (1% BSA, 10% donkey serum, and 0.1% Triton X-100 in DPBS) for 45 minutes. All these steps were carried out at room temperature. Primary antibodies against CD45 (monoclonal, rat; Novus Biologicals, cat. #NB100-77417SS; RRID: AB_1083776; 1:400) and S100 (polyclonal, rabbit; Dako, cat. #Z0311; RRID: AB_10013383; 1:200) were incubated overnight at 4°C in antibody diluent (1% BSA and 0.1% Triton-X in DPBS). The sections were washed in DPBS for 10 minutes and incubated with secondary antibodies (donkey anti-rabbit; Life Technologies, cat. #A31573, RRID: AB_2536183, 1:400 and donkey anti-rat; Thermo Fisher Scientific, cat. #A21208, RRID AB_141709) in antibody diluent for 45 minutes at room temperature. The sections were washed twice in DPBS for 7.5 minutes at room temperature. Finally, nuclei were stained with DAPI (Thermo Fisher Scientific, #D3571) solution (0.5 mg/mL), diluted 1:1,000 in DPBS for 5 minutes, and mounted in ProLong-Diamond Antifade Mountant (Thermo Fisher Scientific, #P36961).

### Multiplexed Immunofluorescence on FFPE Sections

*NRAS;Ink4a* and YUMM5.2 tumors were collected upon mouse sacrifice and placed in 4% formaldehyde solution overnight. Afterward, samples were moved to 70% ethanol and subsequently included in paraffin. Then 5-μm FFPE tissue sections of selected samples were cut and mounted on coverslips.

Prior to staining, all tissue slides were deparaffinized on the Leica BOND RX automated Immunostainer (Leica Microsystems) by heating them for 30 minutes at 60°C, soaking in BOND Dewax Solution at 72°C, and then rehydrating in ethanol. Staining was performed on the same instrument with heat-induced epitope retrieval pretreatments applied at 95°C using BOND Epitope Retrieval (ER) Solutions: citrate-based pH 6.0 ER1 (Leica Biosystems, cat. #AR9961) or EDTA-based pH 9.0 ER2 (Leica Biosystems, cat. #AR9640) according to the manufacturer’s protocol.

Primary antibodies (rabbit anti-PDGFRα, Abcam, cat. #ab203491; goat anti-NKP46, R&D Systems, cat. #AF2225; rabbit anti-CD45, Cell Signaling Technology, cat. #70257S; rabbit anti-CD3ε, Abcam, cat. #ab16669; rabbit anti-SOX10, Abcam, cat. #ab155279; rabbit anti-CD11c, Cell Signaling Technology, cat. #97585S; rat anti-F4/80, Invitrogen, cat. #MF48000; and rat anti-CD8α, Invitrogen, cat. #14-0808-80) were incubated at room temperature for 30 minutes. The antibodies were detected with goat anti–rabbit horseradish peroxidase (HRP; EnVision + System-HRP Labeled Polymer Anti-Rabbit, DAKO, K4003), goat anti–rat HRP (ImmPRESS HRP Anti-Rat IgG, Vector Laboratories, MP-7404), or horse anti–goat HRP (Horse Anti-Goat IgG ImmPRESS; Vector Laboratories, MP-7405) for 15 minutes and visualized using tyramide signal amplification with 10-minute incubations (Opal 6-Plex Detection Kit for Whole Slide Imaging – formerly Opal Polaris 7 Color IHC Automated Detection Kit – Akoya Biosciences, cat. #NEL871001KT). Two different panels were used: (i) NKp46 Opal 520, RRID: AB_355192; CD3ε Opal 570, RRID: AB_443425; CD11c Opal 620, RRID: AB_2800282; CD45 Opal480, RRID: AB_2799780; F4/80 Opal 690, RRID: AB_1500089; SOX10, RRID: AB_2650603; or PDGFRa, RRID: AB_2892065, Opal 780, and (ii) CD45 Opal 520; CD3ε Opal 570; and CD8α Opal 690, RRID: AB_2572860 and SOX10 or PDGFRa Opal 780. Sections were counterstained with DAPI for nuclei visualization and subsequently mounted using ProLong Diamond Antifade Mountant (Invitrogen). All fluorescently labeled slides were scanned on the PhenoImager (formerly Vectra Polaris, Akoya Biosciences) at 40× magnification using appropriate exposure times for whole-slide scanning with the seven-color whole-slide unmixing filters (DAPI + Opal 570/690, Opal 480/620/780, and Opal 520). The analysis of the final qptiff images was performed with QuPath v4.0.

### MC38 Mouse Model

Eight-week-old C57BL/6 mice were injected with 1.5 million MC38 tumor cells. On day 8, 100 μg of PD-1 blocking antibody was given. Next dose was administered on day 11 and mice were sacrificed on day 12. The control group received control antibody. Tumor samples were collected and single-cell suspension was generated. The single-cell suspension was stained with viability dye and then surface marker antibody mixture was applied for 30 minutes at 4°C. The cells were washed and fix/permed overnight. The day after, the cells were subjected to intracellular staining [as described in Song and colleagues (34) in the accompanied article] and samples were acquired on the Cytek Aurora flow cytometry machine.

Collected data were uploaded to web-based software OMIQ, and dimension reduction (Uniform Manifold Approximation and Projection) and clustering (FlowSOM) were applied. To further confirm, two-dimensional flow plots were generated by FlowJo software.

### Genetic Models of CD8 T-cell Depletion

To assess the role of CD8^+^ T cells in ICB response in the *NRAS;Ink4a* model, we developed a murine model, in which CD8^+^ T cells can be specifically depleted by Dyphtheria Toxin A (DTA) administration (Merck, cat. #D0564). To this aim, we crossed the murine model C57BL/6-Tg(Cd8a-cre)1Itan/J (which expressed the Cre recombinase under the control of CD8 promoter and so specifically in CD8^+^ T cells) with the murine model Gt(ROSA)26Sortm1(HBEGF)Awai (in which the diphtheria toxin receptor is floxed). We generated the murine model C57BL/6-Tg(Cd8a-cre)1Itan/J; Gt(ROSA)26Sortm1(HBEGF)Awai, in which CD8^+^ T cells specifically and successfully express DTR and can be depleted by DTA administration. To confirm the depletion, a cohort of seven mice (three control vs. four DTA treated) was treated for 14 days with DTA (0.5 μg/mouse, three times per week, intraperitoneal injection; ref. 52). Afterward, mouse spleen was harvested, red blood cell were lysed, and splenocytes were incubated for 15 minutes on ice with eFluor 780 (1:5,000; Thermo Fisher Scientific) and FcR Blocking Reagent (1:1,000; Miltenyi Biotec). The cells were then washed and incubated for 30 minutes on ice in PBS + 2% FBS with the following antibodies: Brilliant Violet 605 anti–mouse CD45 (1:200; BioLegend, cat. #103140, RRID: AB_2562342), Brilliant Violet 421 anti–mouse CD3ε (1:200; BioLegend, cat. #100336, RRID: AB_11203705), FITC anti–mouse F4/80 (1:200; Invitrogen, cat. #MA5-16628, RRID: AB_2538124), PE/Cyanine7 anti–mouse Ly-6G (1:200; BioLegend, cat. #127618, RRID: AB_1877261), BV711 anti–mouse CD4 (1:200; BD Horizon, cat. #563050, RRID: AB_2737973), and AlexaFluor 700 anti–mouse CD8a (1:200; BD Pharmingen, cat. #557959, RRID: AB_396959, 1:400). After staining, the cells were washed and processed by flow cytometry with BD LSRFortessa X-20 + high-throughput sampler.

To confirm the role of CD8 T-cell depletion in *NRAS;Ink4a* response to ICB response, 2.5 × 10^5^*NRAS;Ink4a* cells were subcutaneously injected into 8- to 12-week-old female C57BL/6-Tg(Cd8a-cre)1Itan/J; Gt(ROSA)26Sortm1(HBEGF)Awai mice. DTA was administered to mice from day 5 after injection (0.5 μg/mouse, three times per week, intraperitoneal injection). To perform NK cell depletion, mice were administered aNK1.1-depleting antibody (Bio X Cell; 100 μg in 200 μL PBS; clone PK136) 3 days before tumor injection and every 5 days after tumor injection. Tumor volume was monitored every other day and calculated using the formula V = (width  ×  length^2^)/2. As soon as tumors reached a volume of ∼20 mm^3^ (indicatively corresponding to a palpable 3 × 3 mm mass), mice were administered either αPD-1 (clone RMP1-14) or IgG2a control isotype (Bio X Cell; 200 μg in 200 μL PBS, twice per week, intraperitoneal injection). Mice were sacrificed (by cervical dislocation) in all the cohorts once the tumor volume in the control cohort reached ∼500 mm^3^.

### Statistical Analysis

For the mouse experiments, sample size is reported in the corresponding figure legend. Violin plots in Fig. 4 and Supplementary Fig. S6 display each single replicate and were analyzed with the Welch-corrected *t* test. To assess progression-free survival, the amount of time needed for the tumor volume to exceed 300 mm^3^ was computed (log-rank Mantel–Cox test).

### Data Availability

Raw sequencing data of all human scRNA-seq (EGAD00001009291) are available the European Genome-phenome Archive under the study number EGAS00001006488 after approval by the UZ Leuven–VIB data access committee as it contains sensitive personal data (according to European GDPR law). Processed data of the immune cells from both time points as well as ShinyApp link are available at KU Leuven Research Data Repository: https://doi.org/10.48804/GSAXBN. Raw sequencing data of mouse scRNA-seq are available in the Gene Expression Omnibus portal under the accession number GSE292854. Spatial transcriptomics data are available upon request. Code used in this article is deposited at https://github.com/orgs/MarineLab/.

## Supplementary Material

Supplemental Table S1 Characteristics of patients for (A) scRNA-seq, (B) MILAN mIF samples, (C) FLOW cytometry of peripheral NK cells.

Supplemental Table S2 Discriminatory marker genes of initial immune cells clusters.

Supplemental Table S3 Discriminatory marker genes of clusters obtained after subclustering of CD8+ T and NK cells.

Supplemental Table S4 Discriminatory marker genes of clusters obtained after subclustering od Myeloid cells.

Supplemental Table S5 Discriminatory marker genes of clusters obtained after subclustering of Macrophages.

Supplemental Table S6 Discriminatory marker genes of clusters obtained after subclustering of DCs and Monocytes.

Supplemental Table S7 Discriminatory marker genes of all immune clusters obtained by summarising unsupervised Louvain clustering and subclustering of myeloid, and CD8+T/NK cells.

Supplemental Table S8 List of antibodies used for the MILAN experiment.

Supplemental Table S9 List of probes used for the Xenium experiment.

Supplemental Table S10 Discriminatory marker genes of clusters obtained after clustering of all immune cells from the scRNA-seq mouse data.

Supplemental Table S11 Discriminatory marker genes of clusters obtained after clustering of all immune cells from the CITE-seq mouse data (NK cells = cluster 11).

Supplemental Table S12 List of antibodies used for the FACS analysis of the peripheral NK cells.

Supplemental Table S13 List of antibodies used for the CITE-seq mouse experiment.

Supplemental Figures S1-S10 Supplemental Figure S1 shows the identification and characterization of major immune cell populations and states across both timepoints, using UMAP visualization, unsupervised clustering, and marker gene expression profiling. Supplemental Figure S2 shows the distribution of immune cell types across samples, timepoints, and response groups, as well as the lack of association between metastatic site and response to immune checkpoint blockade. Supplemental Figure S3 shows the comparison of immune cell type proportions across timepoints and response groups, and validates the association between NK cells and lack of response using multiple independent melanoma and breast cancer cohorts. Supplemental Figure S4 shows flow cytometry-based characterization of peripheral blood NK (PBNK) cells, including gating strategy, subset distribution, and expression of cytotoxic markers across treatment timepoints and response groups. Supplemental Figure S5 shows the relationship between immune cell composition and tumor-infiltrating lymphocyte (TIL) patterns, CD8⁺ T cell density across regions and response groups, and spatial localization of NK cells in representative lesions from responders and non-responders. Supplemental Figure S6 shows spatial transcriptomic analysis of the tumor microenvironment (TME) using Xenium, including clustering and marker gene expression of TME cells, spatial localization of NK cells in cold and immune-excluded tumors, and neighborhood enrichment patterns of cell types in cold tumors. Supplemental Figure S7 shows the spatial distribution of NK cells in murine melanoma models (YUMM5.2 and NRAS;Ink4a) using immunofluorescence, H&E, and multiplex immunostaining, highlighting NK cell localization relative to melanoma and immune markers across treatments. Supplemental Figure S8 shows the impact of NK cell and PD-1 blockade on YUMM1.7 tumor growth in vivo, supported by histological and multiplex immunostaining to assess immune infiltration across treatment groups. Supplemental Figure S9 shows single-cell and multiplex immunofluorescence analyses of the tumor microenvironment in NRAS;Ink4a and YUMM5.2 murine tumors across treatment conditions, highlighting immune and malignant cell cell composition, and CD8⁺ T cell abundance in the spleen after depletion of this cell type. Supplemental Figure S10 shows immune profiling of NRAS;Ink4a and MC38 tumors under control and anti–PD-1 conditions using CITE-seq, flow cytometry, and multiplex immunofluorescence, highlighting NK cell dynamics, immune composition, and spatial organization across treatment cohorts.
