# Supplementary material for: Cytotoxic NK Cells Impede Response to Checkpoint Immunotherapy in Melanoma with an Immune-Excluded Phenotype
Source: Cancer Discov. 2025 Jun 18;15(9):1819–34. doi: 10.1158/2159-8290.CD-24-1208 (PMC12409281; doi:10.1158/2159-8290.CD-24-1208)
Supplement: Supplemental Figures S1-S10 — Supplemental Figure S1 shows the identification and characterization of major immune cell populations and states across both timepoints, using UMAP visualization, unsupervised clustering, and marker gene expression profiling. Supplemental Figure S2 shows the distribution of immune cell types across samples, timepoints, and response groups, as well as the lack of association between metastatic site and response to immune checkpoint blockade. Supplemental Figure S3 shows the comparison of immune cell type proportions across timepoints and response groups, and validates the association between NK cells and lack of response using multiple independent melanoma and breast cancer cohorts. Supplemental Figure S4 shows flow cytometry-based characterization of peripheral blood NK (PBNK) cells, including gating strategy, subset distribution, and expression of cytotoxic markers across treatment timepoints and response groups. Supplemental Figure S5 shows the relationship between immune cell composition and tumor-infiltrating lymphocyte (TIL) patterns, CD8⁺ T cell density across regions and response groups, and spatial localization of NK cells in representative lesions from responders and non-responders. Supplemental Figure S6 shows spatial transcriptomic analysis of the tumor microenvironment (TME) using Xenium, including clustering and marker gene expression of TME cells, spatial localization of NK cells in cold and immune-excluded tumors, and neighborhood enrichment patterns of cell types in cold tumors. Supplemental Figure S7 shows the spatial distribution of NK cells in murine melanoma models (YUMM5.2 and NRAS;Ink4a) using immunofluorescence, H&E, and multiplex immunostaining, highlighting NK cell localization relative to melanoma and immune markers across treatments. Supplemental Figure S8 shows the impact of NK cell and PD-1 blockade on YUMM1.7 tumor growth in vivo, supported by histological and multiplex immunostaining to assess immune infiltration across treatment group [file cd-24-1208_supplemental_figures_s1-s10_suppsf1.pdf]

Supplemental figures

Supplemental Figure S1

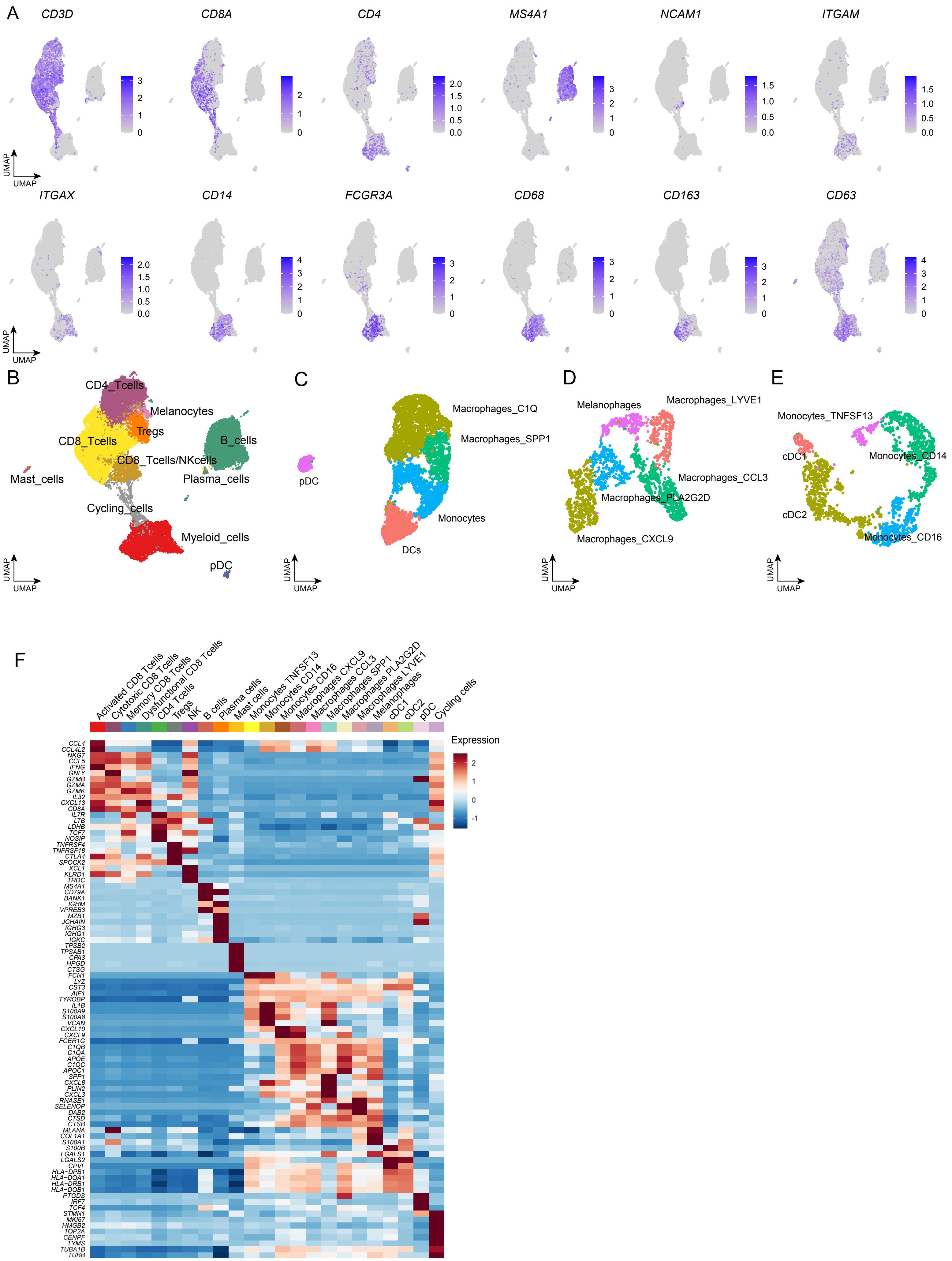

Supplemental Figure S1:

A, Uniform Manifold Approximation and Projection (UMAP) of the common marker genes of the main immune cell types including samples from both timepoints**.**

B, UMAP of the main immune cell types identified by unsupervised Louvain clustering including samples from both timepoints.

C-E, UMAP of subclustered Myeloid cells together (C), Macrophages C1Q (**D**) and Dendritic cells with monocytes (**E**) including samples from both timepoints.

F, Heatmap representing the top 10 discriminatory marker genes of all immune cell types and states identified by summarising unsupervised Louvain clustering and subclustering of myeloid, and $CD8^{+}$T/NK cells including samples from both timepoints.

Supplemental Figure S2

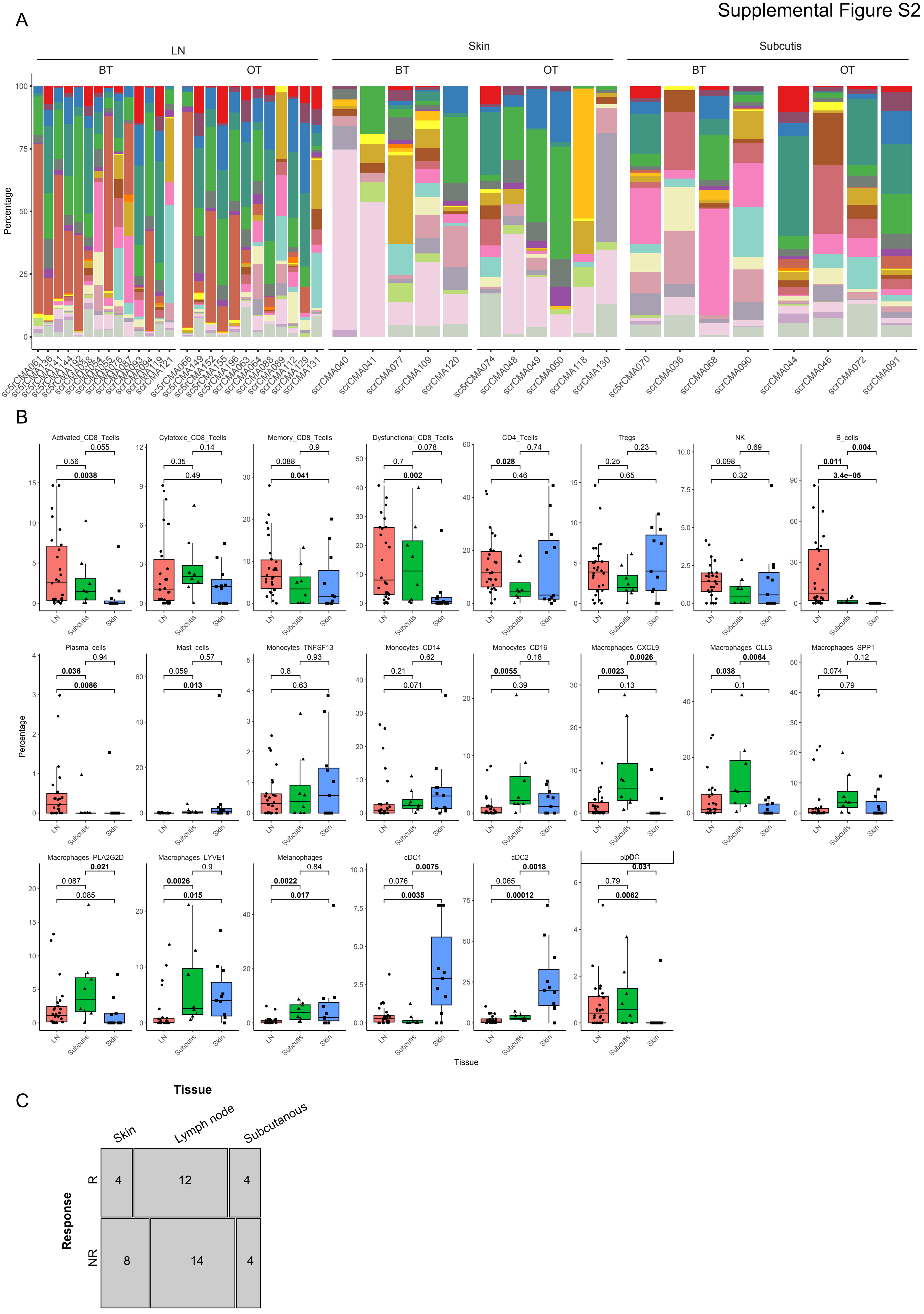

Supplemental Figure S2:

A, Proportions of the immune types of the total immune cell number presented in the figure 1D in each sample grouped by metastatic location and timepoint (BT: Before Treatment, OT: On Treatment).

B, Percentages of identified immune cell types out of all immune cells across before treatment (BT) and on treatment (OT) samples and both response groups (two-sided Wilcoxon test, bold = $p<0.05$).

C, Mosaic plot representing lack of association of the metastatic site and response to ICB.

Supplemental Figure S3

A

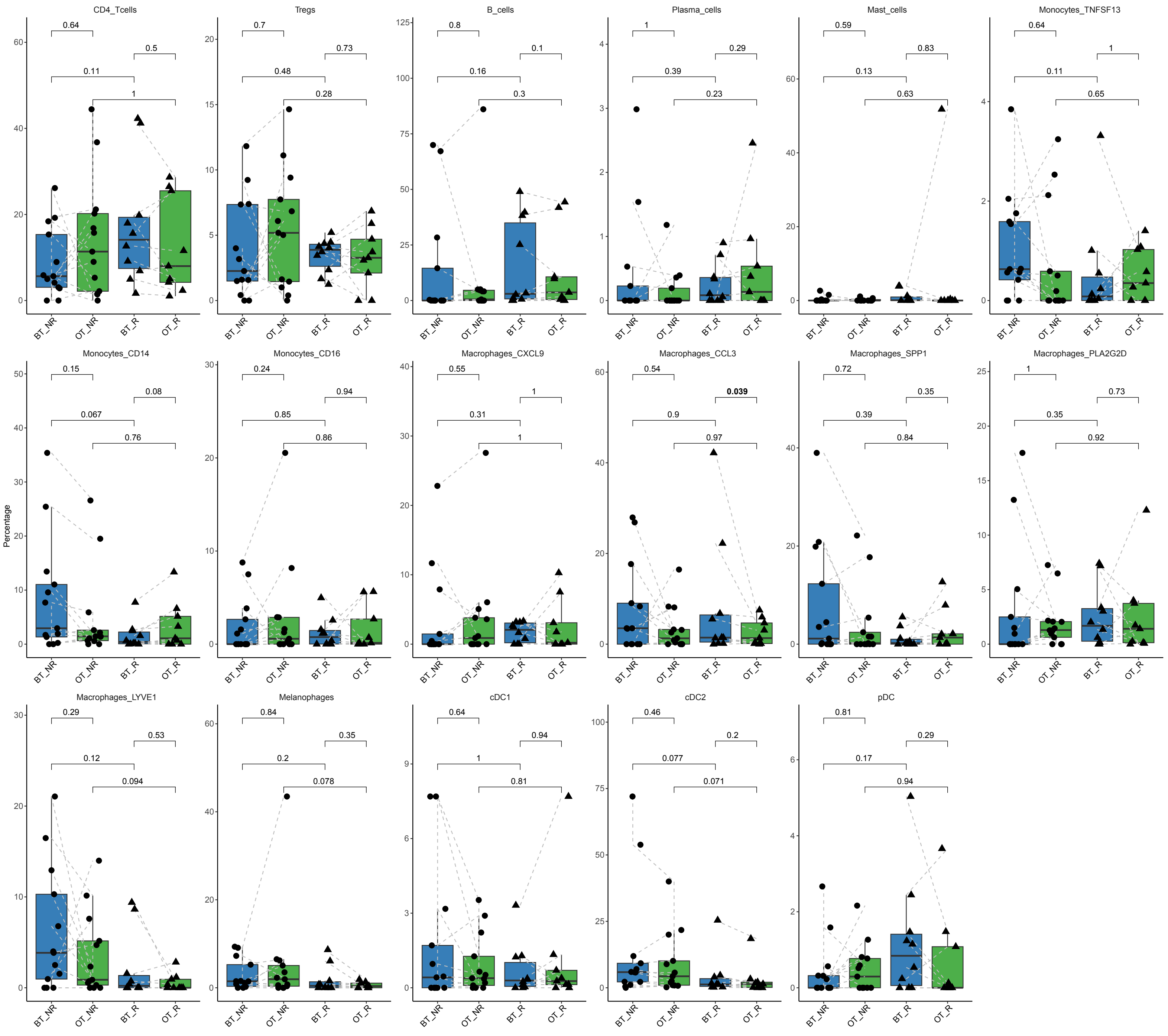

B

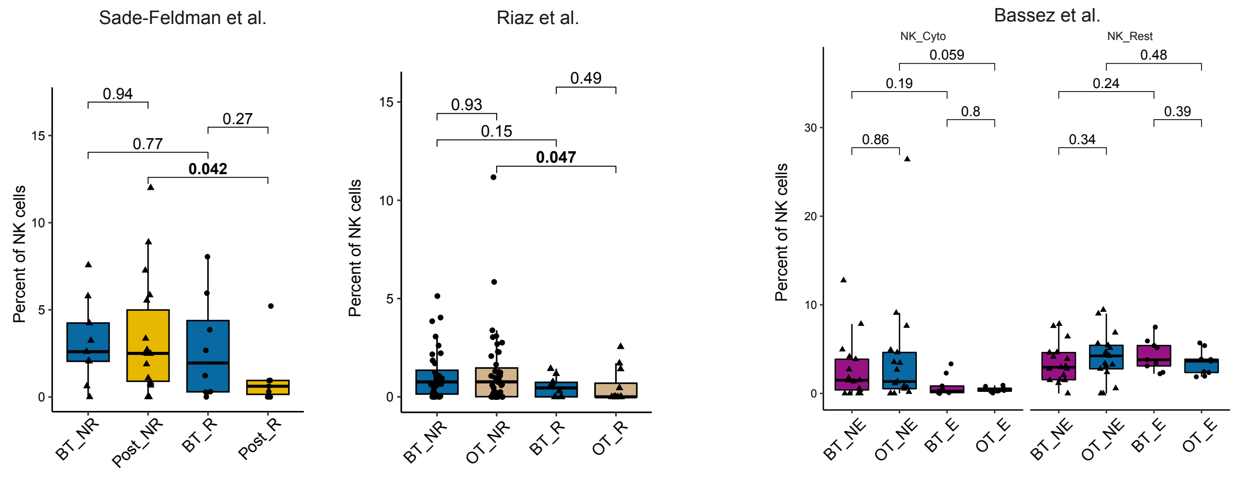

Supplemental Figure S3:

A, Percentages of identified immune cell types out of all immune cells across before treatment (BT) and on treatment (OT) samples and both response groups (two-sided Wilcoxon test, bold = $p<0.05$).

B, Validation of NK cells (identified in the inhouse cohort) association with lack of response in single cell RNA-seq melanoma cohort(1) identified based on NK cell score acquired from the inhouse cohort (left); in bulk RNA-seq melanoma cohort identified based on CIBERSORTx (middle); and in the single cell RNA-seq breast cancer cohort identified as in the original study (right)(2). Two-sided Wilcoxon test, bold text points to significant differences, $p<0.05$.

Supplemental Figure S4

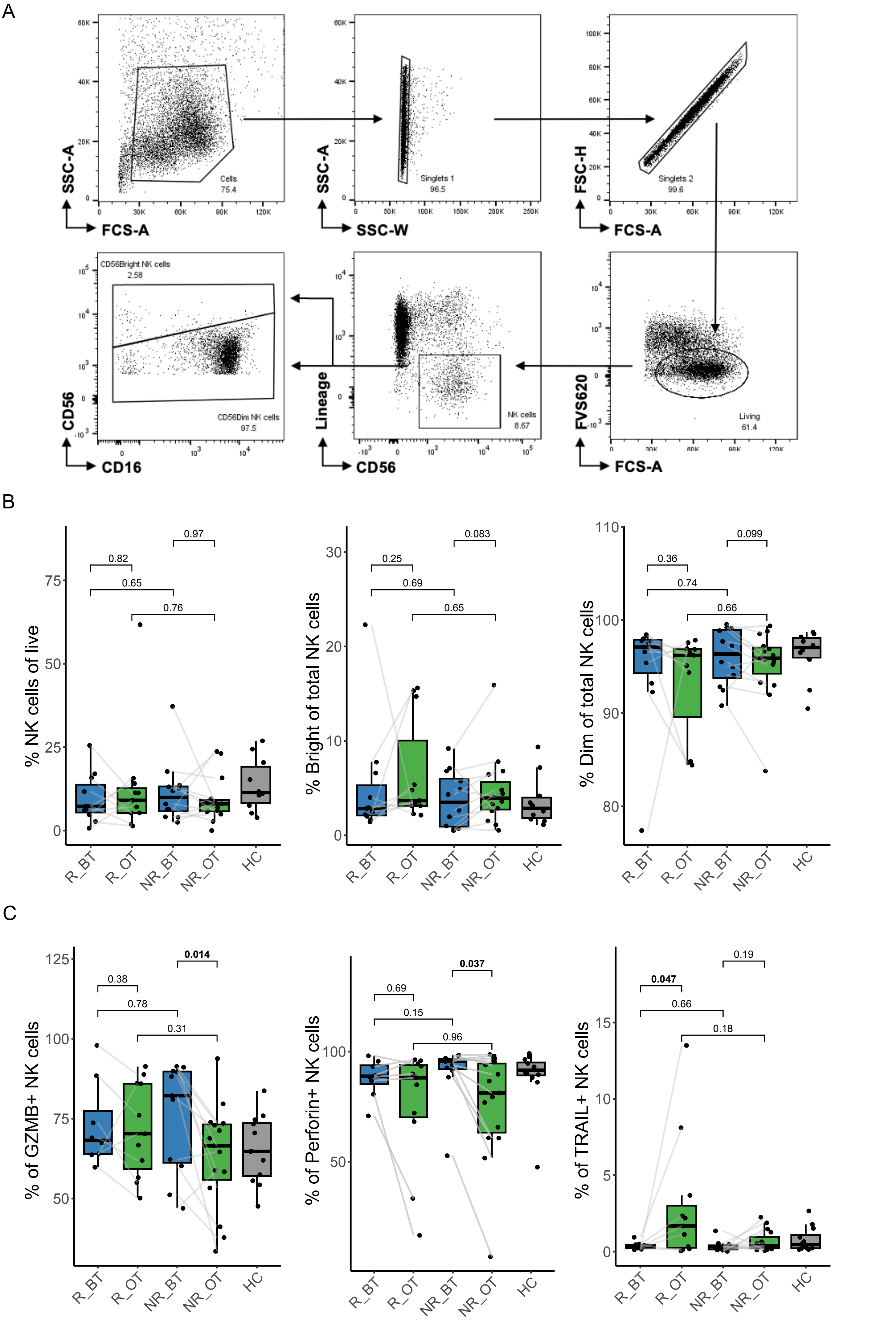

Supplemental Figure S4:

A, Representative gating strategy of PBNKs in PBMCs is shown. PBNKs are defined as CD3- CD14- CD19- CD123- CD56+.

B, FACS based percentages of living PBNK (CD3- CD14- CD19- CD123- CD56+) cells from total living PBMCs (left), CD56dim PBNK cells (CD3- CD14- CD19- CD123- CD56dim CD16+) from total PBNK cells (middle) and CD56bright PBNK cells (CD3- CD14- CD19- CD123- CD56bright CD16-) from total PBNK cells (right) in BT-NR, BT-R, OT-R and OT-NR patients.

C, FACS based percentages of Granzyme B+ PBNK cells (left), Perforin+ PBNK cells (middle) and TRAIL+ PBNK cells (right) in BT-NR, BT-R, OT-R and OT-NR patients.

Two-sided Wilcoxon test, bold = $p<0.05$

BT, Before Treatment; OT, On Treatment; NR, non-responder; R, responder

Supplemental Figure S5

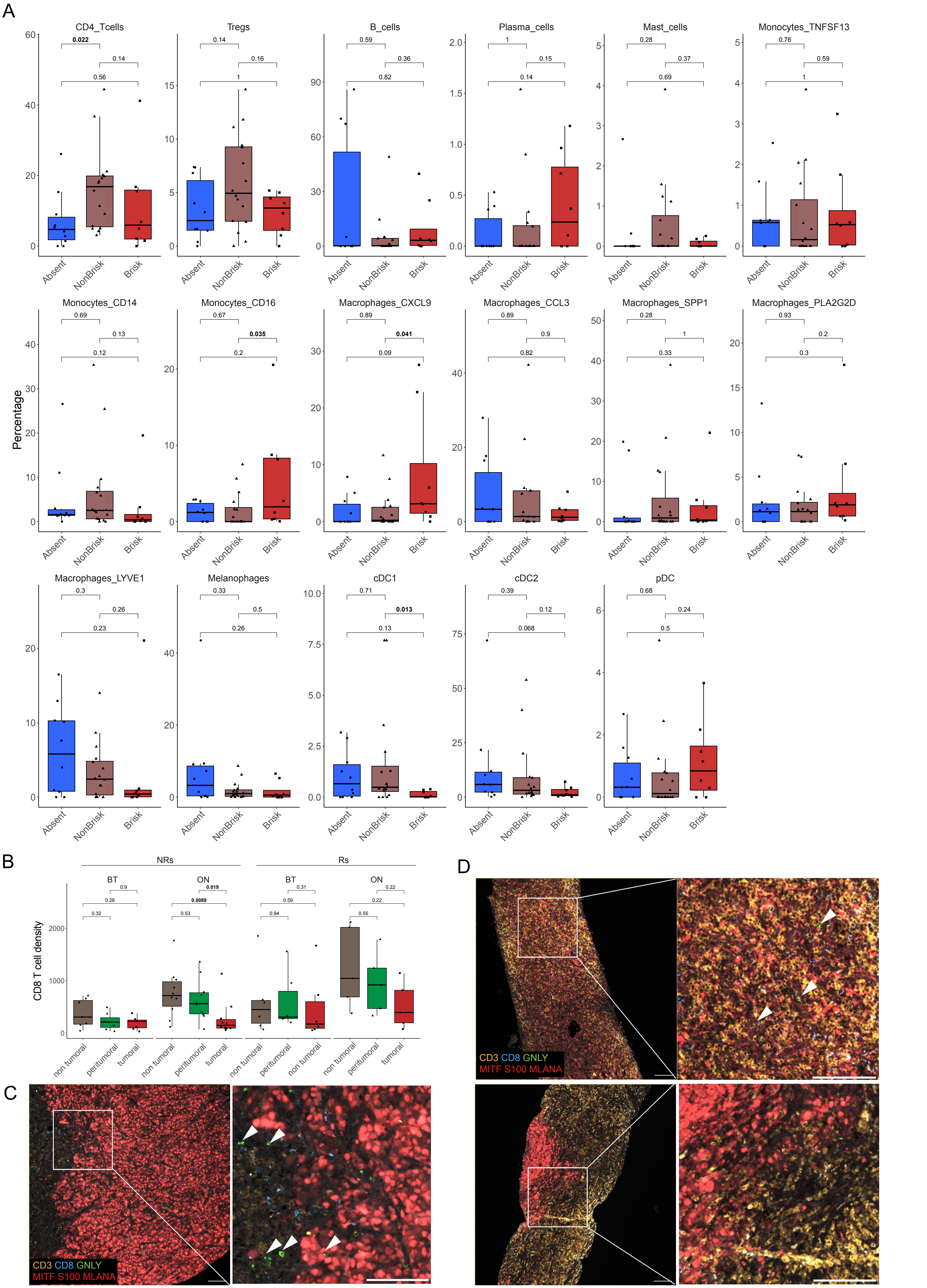

Supplemental Figure S5:

A, Percentages of identified immune cell types out of all immune out of all immune cells plotted among three tumor infiltrating lymphocytes (TILS): “absent” (cold), “non-brisk” (excluded) and “brisk” (hot) assessed by pathologist. Two-sided Wilcoxon test, bold = $p<0.05$.

B, Density per square milimiter (sqmm) of CD8T cells compared between three different tumoral regions, response and timepoint, two-sided Wilcoxon test, bold = $p<0.05$.

C, Example of a lesion from a non-responder where NK cells were detected at the rim of the tumor. Green = GNLY, red = melanoma, orange = CD3, blue = CD8 and arrows point NK cells.

D, Example of two lesions from responders where NK cells intermingled with other immune cells (top) or were not detected at all (bottom). Green = GNLY, red = melanoma, orange = CD3, blue = CD8 and arrows point NK cells.

Supplemental Figure S6

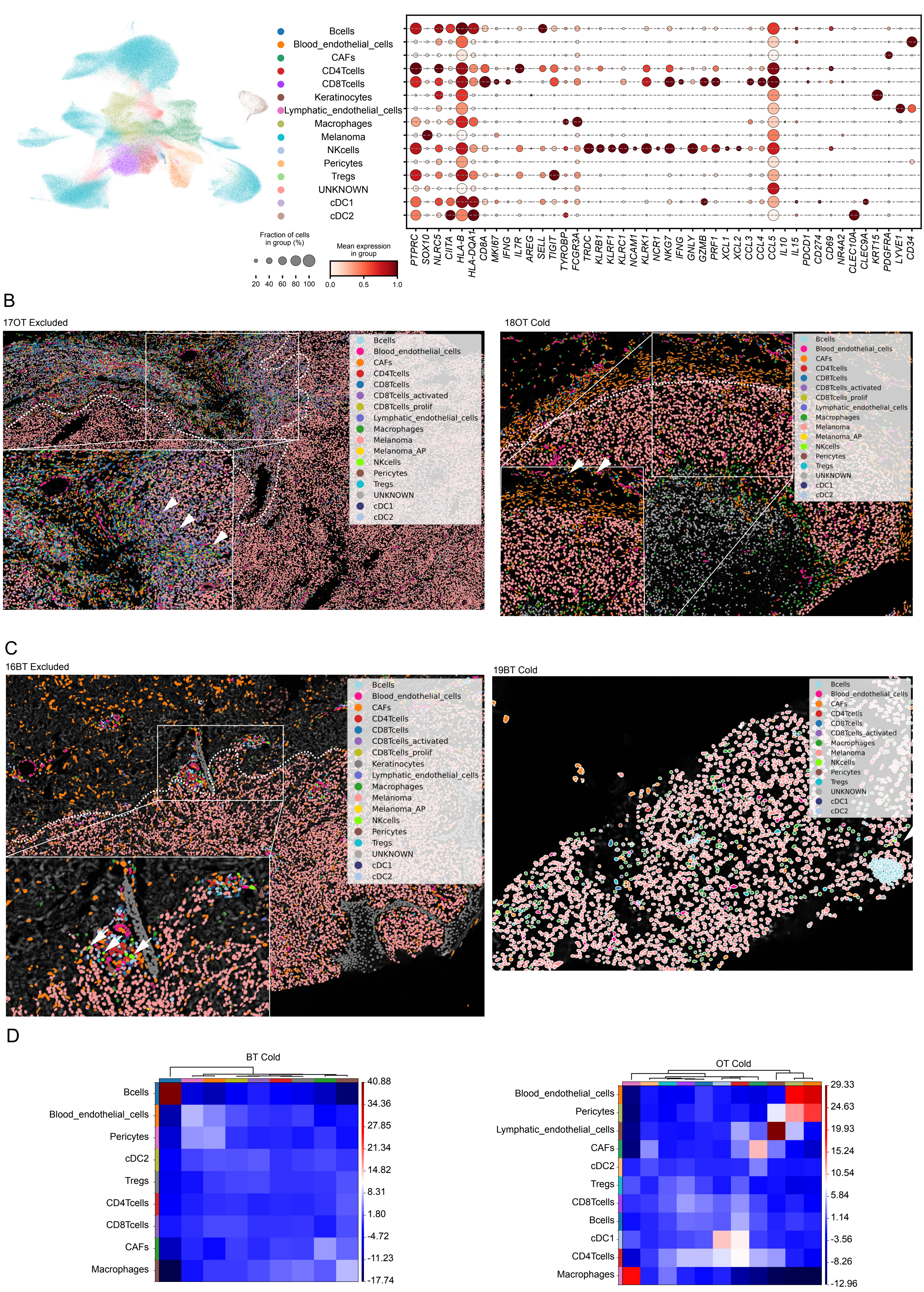

Supplemental Figure S6:

A, UMAP plot of all TME cells from all samples used for the Xenium experiment. Clusters were identified using Leiden clustering (left)**;** Dotplot of discriminatory genes identified by spatial transcriptomics (Xenium) between TME cells and NK related genes across annotated TME cells grouping all samples together (right). Size of the dot represents the number of cells expressing the gene and colour reflects the expression level.

B, Spatial plots of all identified cells from examples of immune excluded (left) and cold (right) tumors from lymph node metastasis from NR OT. Arrows point towards NK cells, and the dashed line represents tumor boarder.

C, Spatial plots of all identified cells from examples of immune excluded (left) and cold (core needle biopsy, right) tumors from lymph node metastasis from NR BT. Arrows point towards NK cells, and the dashed line represents tumor boarder.

D, Heatmaps of neighbourhood enrichment of cell types/states identified using Xenium spatial transcriptomics for cold tumors. Cells which abundance was <5, as well as cells annotated as either UNKNOWN or keratinocytes were removed for the neighbourhood analyses. The scale represents the z-scores from a permutation test, indicating how frequently each pair of cell types was observed as neighbours compared to a randomly permuted spatial distribution.

Supplemental Figure S7

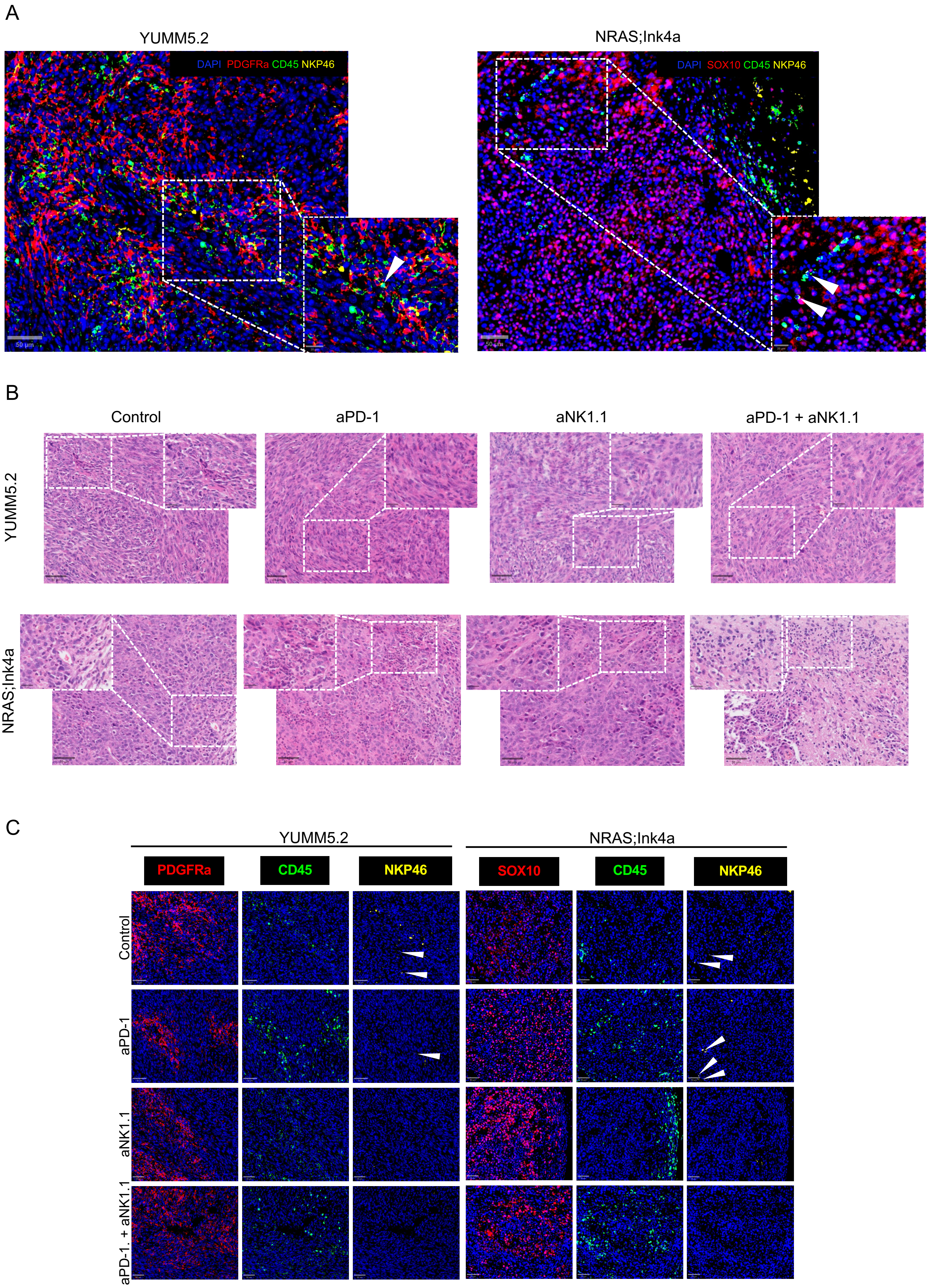

Supplemental Figure S7:

A, Immunofluorescence of YUMM5.2 (left panel) and NRAS;Ink4a tumors (right panel). Tumors were stained for a melanoma marker (PDGFRa for YUMM5.2 and SOX10 for NRAS;Ink4a) and for CD45 and NKP46 to map the NK cell distribution. Counterstaining was performed with DAPI. Arrows indicate NK cells.

B, Haematoxylin and eosin staining of YUMM5.2 (top panel) and NRAS;Ink4a tumors (bottom panel) across treatments.

C, Single images of OPAL 6plex staining of YUMM5.2 (left panel) and NRAS;Ink4a tumors (right panel). Tumors were stained for a melanoma marker (PDGFRa for YUMM5.2 and SOX10 for NRAS;Ink4a), for CD45 to map the immune infiltration, and for NKP46 to mark NK cells. Arrows indicate NK cells.

A

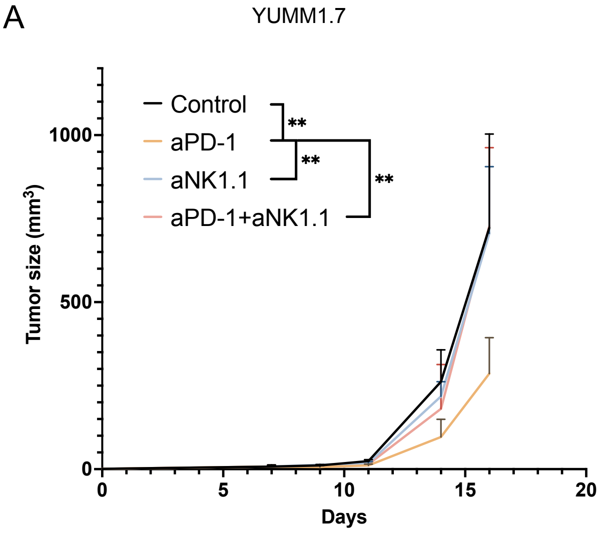
YUMM1.7
Control
aPD-1
aNK1.1
aPD-1+aNK1.1
**
**
**
Tumor size (mm³)
1000
500
0
0
5
10
15
20
Days

B

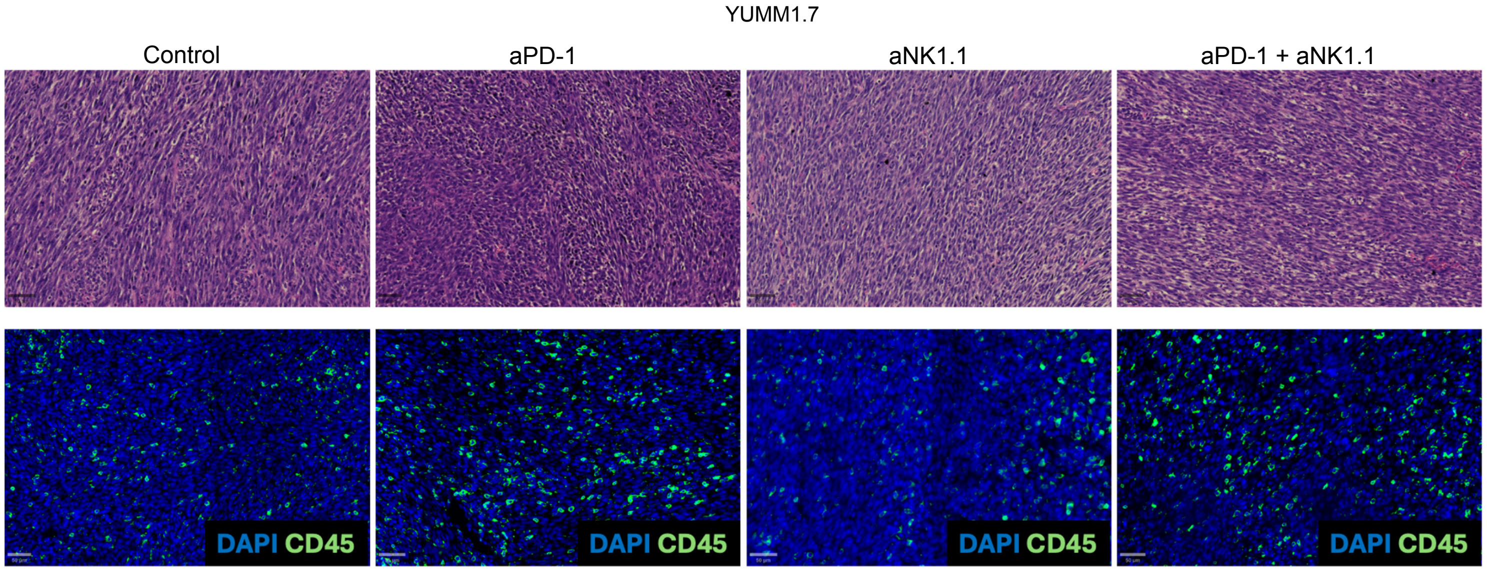
YUMM1.7
Control
aPD-1
aNK1.1
aPD-1 + aNK1.1
DAPI CD45
DAPI CD45
DAPI CD45
DAPI CD45

Supplemental Figure S8:

A, Growth curves of YUMM1.7 tumors (bottom left) *in vivo* ($n \geq 5$ per cohort (control, aPD-1, aNK1.1, aPD-1+aNK1.1); Welch-corrected t-test was performed on the doubling time of tumors, upon exponential fitting; $^{**}p<0.01$).

B, Haematoxylin and eosin staining of YUMM1.7 tumors across cohorts (top panel). Single images of OPAL 6plex staining of YUMM1.7 (bottom panel). Tumors were stained for CD45 to map the immune infiltration.

Supplemental Figure S9

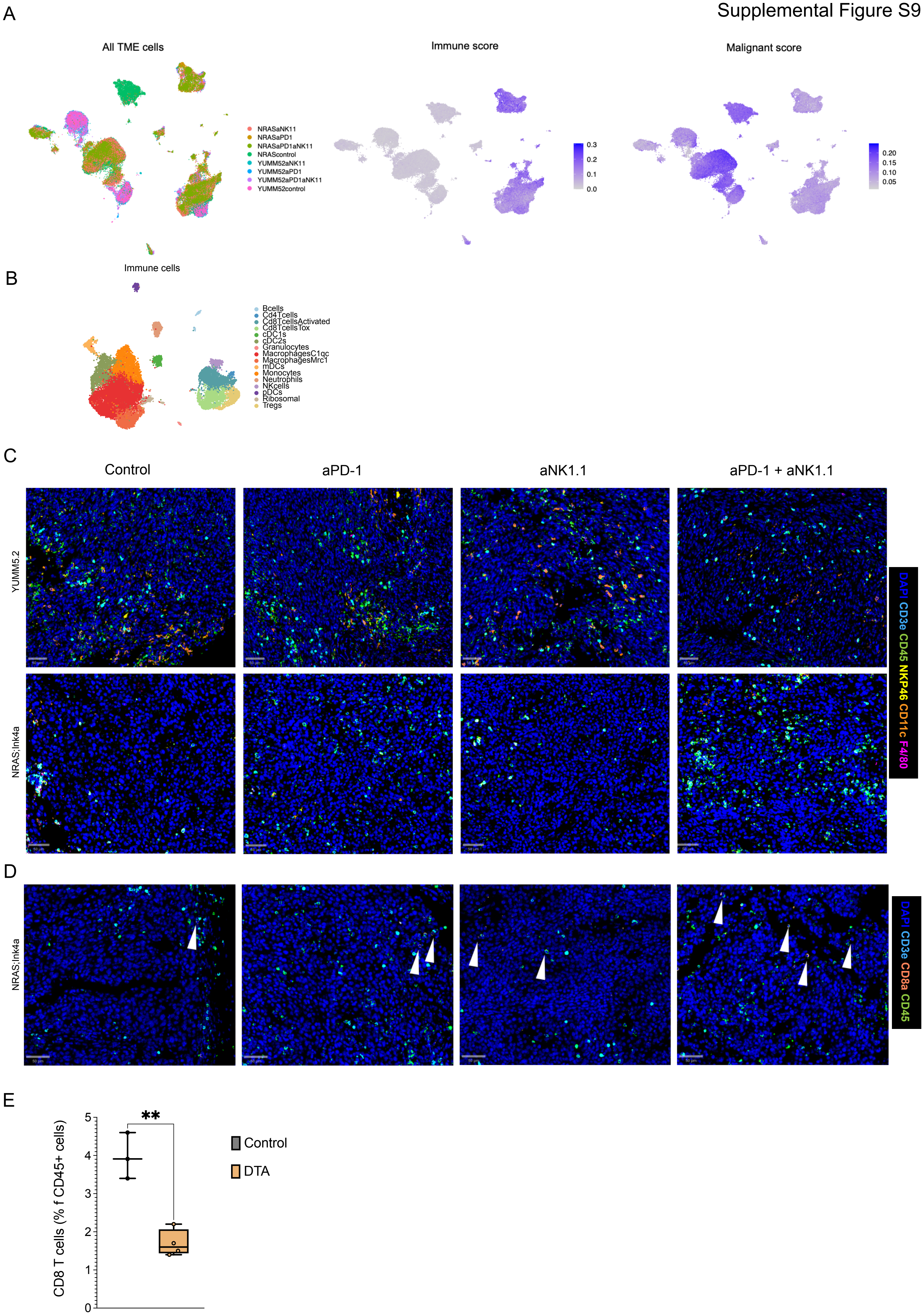

Supplemental Figure S9:

A, UMAP of all annotated tumor microenvironment cells from the NRAS;Ink4a and YUMM5.2 tumors from all conditions (left), UMAP coloured by Immune (middle) and Malignant score (right); based on the scRNA-seq experiment.

B, UMAP of all annotated immune cells from the NRAS;Ink4a and YUMM5.2 tumors from all conditions (top).

C, OPAL 6plex immunofluorescence of YUMM5.2 (top panel) and NRAS;Ink4a tumors (bottom panel) across cohorts. Tumors were stained for CD45 (immune cells), CD11c (dendritic cells), NKP46 (NK cells), F4/80 (macrophages), and CD3ε (T cells). Tumors were stained for a melanoma marker (PDGFRa for YUMM5.2 and SOX10 for NRAS;Ink4a; not shown). Counterstaining was performed with DAPI.

D, OPAL 6plex immunofluorescence of NRAS;Ink4a tumors across cohorts. Tumors were stained for CD45 (immune cells), CD3ε (T cells), and CD8α (CD8 T cells). Counterstaining was performed with DAPI. Arrows indicate CD45+CD3ε+CD8α+ T cells. Arrows indicate CD8 T cells.

E, FACS based quantification of $CD8^+$ T cells in the spleen of C57BL/6-Tg(Cd8a-cre)1Itan/J; Gt(ROSA)26Sortm1(HBEGF)Awai mice. $CD8^+$ T cell ($CD45^+$, $F4/80^-$; $LY6G^-$; $CD3^+$; $CD8^+$) was computed out of all the $CD45^+$ cells ($n \geq 3$ per cohort; Welch-corrected t-test; $^{**}p<0.01$).

Supplemental Figure S10

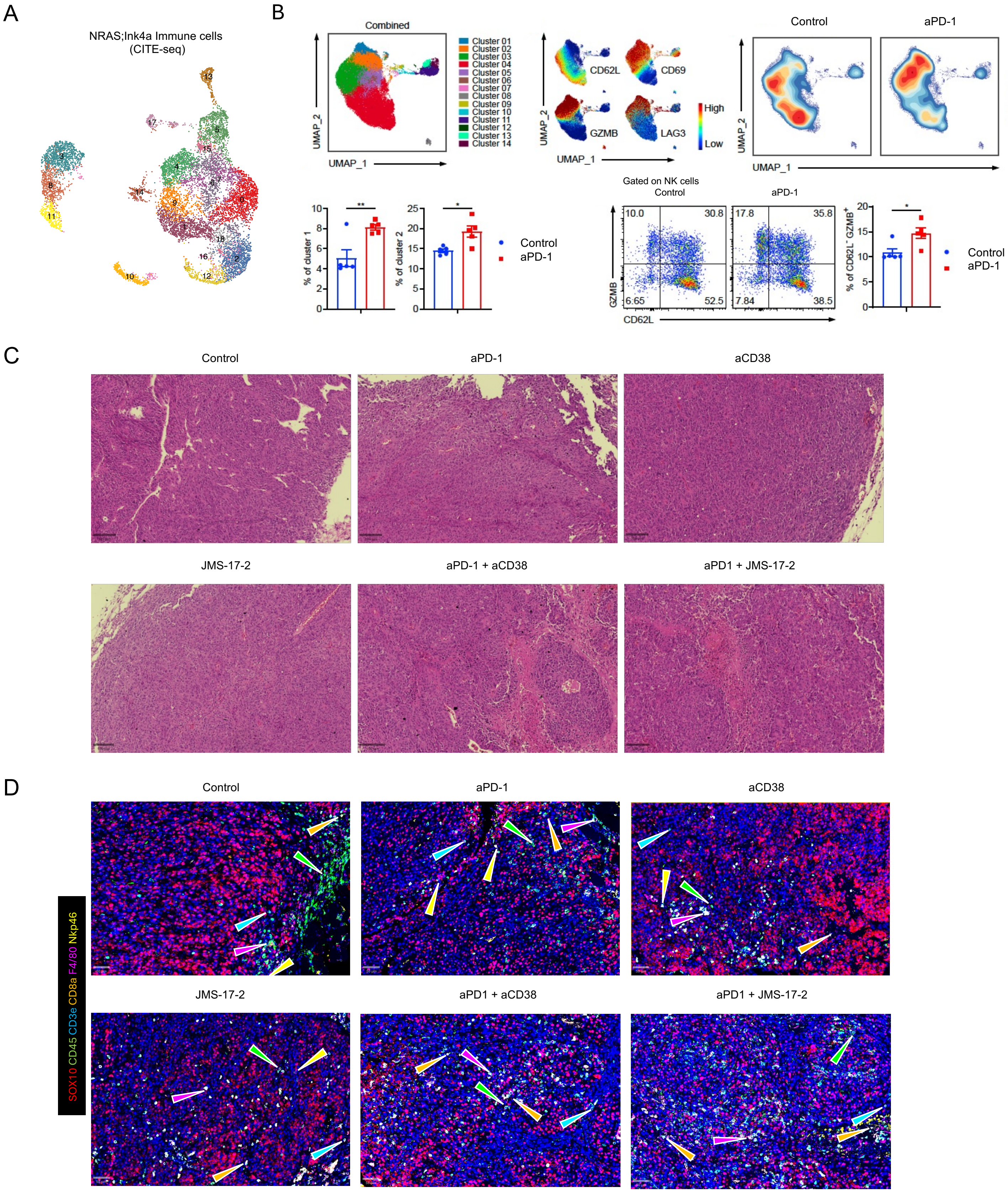

Supplemental Figure S10:

A, UMAP of all annotated immune cells from the NRAS;Ink4a control and aPD-1 condition; based on the CITE-seq experiment.

B, UMAP dimension reduction and FlowSOM clustering were applied to visualize NK cells from control and aPD-1 treated mouse tumor samples (MC38 model; top left). Key marker expressions were overlaid onto UMAP space (middle top), and contour plots were generated to visualize population dynamics (right top). Frequencies of cluster 1 and 2 were plotted (bottom left). Flow cytometry 2D plots were generated to assess frequencies of $CD62L^{-}$ $GZMB^{+}$ NK cell population (student t-test was used for bottom results; $^{*}p<0.05$, $^{**}p<0.01$).

C, Hematoxylin and eosin staining of NRAS;Ink4a tumors across multiple cohorts.

D, Multiplexed immunofluorescence of NRAS;Ink4a tumors. Tumors were stained forSOX10 (melanoma cells), CD45 (immune cells), CD3ε (T cells), CD8α (CD8 T cells), F4/80 (macrophages), and NKP46 (NK cells). Counterstaining was performed with DAPI. Arrows indicate cell types with matched marker color (e.g., yellow for NKP46+ NK cells).

References:

1. Sade-Feldman M, Yizhak K, Bjorgaard SL, Ray JP, de Boer CG, Jenkins RW, et al. Defining T Cell States Associated with Response to Checkpoint Immunotherapy in Melanoma. Cell. Cell Press; 2018;175:998-1013.e20.
2. Bassez A, Vos H, Van Dyck L, Floris G, Arijs I, Desmedt C, et al. A single-cell map of intratumoral changes during anti-PD1 treatment of patients with breast cancer. Nat Med. 2021;27:820–32.
